# Fiber Selection for Reinforced Additive Manufacturing

**DOI:** 10.3390/polym13142231

**Published:** 2021-07-07

**Authors:** Ivan Philip Beckman, Christine Lozano, Elton Freeman, Guillermo Riveros

**Affiliations:** Information Technology Laboratory, U.S. Army Engineer Research and Development Center, Vicksburg, MS 39180, USA; christine.m.lozano@usace.army.mil (C.L.); elton.l.freeman.civ@mail.mil (E.F.); guillermo.a.riveros@usace.army.mil (G.R.)

**Keywords:** short cut, chopped strands, staple fibers, cellulose, keratin, tenacity, carbon fiber, synthetic fiber, ceramic fiber, megaYuri

## Abstract

The purpose of this review is to survey, categorize, and compare the mechanical and thermal characteristics of fibers in order to assist designers with the selection of fibers for inclusion as reinforcing materials in the additive manufacturing process. The vast “family of fibers” is described with a Venn diagram to highlight natural, synthetic, organic, ceramic, and mineral categories. This review explores the history and practical uses of particular fiber types and explains fiber production methods in general terms. The focus is on short-cut fibers including staple fibers, chopped strands, and whiskers added to polymeric matrix resins to influence the bulk properties of the resulting printed materials. This review discusses common measurements for specific strength and tenacity in the textile and construction industries, including denier and tex, and discusses the proposed “yuri” measurement unit. Individual fibers are selected from subcategories and compared in terms of their mechanical and thermal properties, i.e., density, tensile strength, tensile stiffness, flexural rigidity, moisture regain, decomposition temperature, thermal expansion, and thermal conductivity. This review concludes with an example of the successful 3D printing of a large boat at the University of Maine and describes considerations for the selection of specific individual fibers used in the additive manufacturing process.

## 1. Introduction

Fused Filament Fabrication (FFF), also known as Fused Deposition Modeling (FDM), is a three-dimensional printing or additive manufacturing technology that is rapidly advancing and developing [1]. The FDM technique extrudes a melted resin through a nozzle that traces an object’s cross section, depositing the resin onto the printed object [1]. The thermal characteristics of thermoplastic polymers make them popular feedstock materials for FFF, based on their ability to melt, flow through the extrusion nozzle, attach to the desired part, cool, and rapidly harden into the desired shape [1]. However, the resulting strength, stiffness, and rigidity of printed objects is considered a limiting factor for the more widespread adoption of FDM [1]. The insertion of particles or fibers into printed materials is an attempt to improve the chemical, mechanical, thermal, and electrical properties of the resulting composites. Fiber Reinforced Additive Manufacturing (FRAM) is a relatively new and rapidly advancing process for the production of components and parts in many industries [2]. The advantages of FDM, which include rapid set up time and easy modification of designs, can significantly benefit from the inclusion of fibers into printed composite materials in order to tailor the mechanical properties of strong and lightweight parts [2].

Throughout this review, fibers will be considered in terms of their usefulness in reinforcing polymeric matrix resins which are commonly used in additive manufacturing. There are currently many commercially available polymeric resins with short-cut reinforcing fibers, with several examples listed in Table 1 [3]. Carbon and glass fibers are the most common choice today, while Kevlar and basalt fibers have been printed as well [1,4]. However, there is an enormous range of fibers that could also be explored for inclusion in additive manufacturing materials. The U.S. Customs and Border Protection agency maintains a list of 1200 fiber trade names that represent 43 generic fiber categories, acknowledging that the list is neither exhaustive nor complete [5]. The purpose of this review is to survey all types of fibers and compare the mechanical and thermal characteristics of particular fibers in order to assist designers in the selection of reinforcing materials that might be appropriate for reinforcing fibers for FRAM.

## 2. Characteristics of Fibers

Fibers are distinguished from other materials added to composites, such as particles or powders, based on their aspect ratio. Fibers are defined as slender and greatly elongated natural and synthetic filaments with a diameter or cross-sectional thickness less than 250 μm and an aspect ratio generally greater than 100:1 [7]. Fibers can be inserted as reinforcements into FDM, either as long continuous fibers or short-cut fibers. Long continuous fibers are carefully designed and placed within composite materials for specific reinforcing purposes, while short-cut fibers are mixed into the resin melt for semirandom placement to improve the bulk material properties of the printed object. Short-cut fibers can be further classified as staple fibers, chopped strands, or whiskers. Staple fibers have lengths from 10 to 400 mm; based on their individual wavy nature, they are well suited for spinning together into yarn [7]. Chopped strands are fibers that have been cut to various lengths from 3 to 50 mm, typically for inclusion in composite materials [7]. Whiskers are tiny fibers only 1 to 2 mm in length and 1 to 2 μm in diameter which have very high strength based on very few defects in their crystalline structure [7]. Despite sometimes having aspect ratios below 100:1, chopped strands and whiskers are still considered fibers based on their shape and nature. Metal filaments with diameters greater than 100 μm are generally considered wires, while those with diameters less than 100 μm are considered fibers.

**Figure 1:**
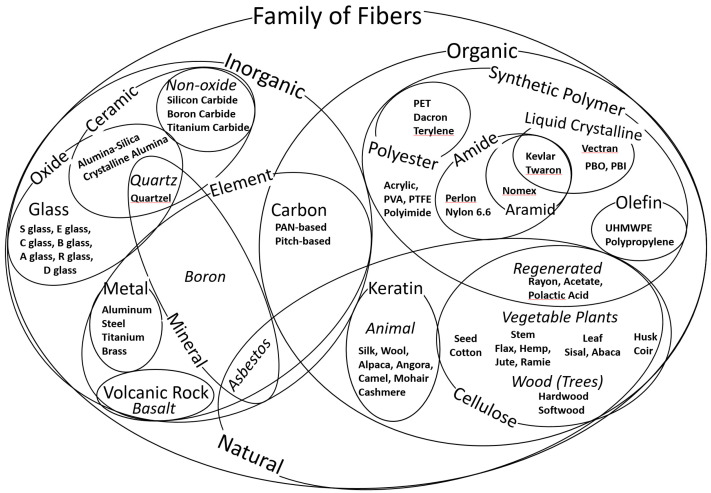
Family of Fibers.

There are many methods to classify and categorize fibers for comparison. The textile institute publishes a diagram of textile fiber types which separates fibers into natural and man-made categories [8]. Since there are additional nontextile fibers that may be appropriate for additive manufacturing, the generic phrase “family of fibers” is used throughout this review to encompass all categories of fibers. A classification for the global family of fibers using organic and inorganic categories as the initial delineation is shown in Figure 1 below and Table A1 in Appendix A.

The categories intersect and overlap, demonstrating the difficulty in classifying fibers. Organic and inorganic fibers may be distinguished from each other by the presence of carbon compounds. Nonetheless, carbon fibers are shown in Figure 1 in both categories, as they are sometimes considered to belong to either category. Section 2 of this review describes the characteristics of organic and inorganic fibers within categories of natural, cellulose, regenerated cellulose, keratin, synthetic polymer, carbon, ceramic, oxide, nonoxide, quartz, glass, mineral, volcanic rock, and metal fibers. The categories and subcategories overlap, as depicted in the Venn diagram in Figure 1. An explanation of the basic terminology used throughout this review is included in Appendix B.

### 2.1. Natural Fibers

Aside from the primary classification of organic and inorganic, a secondary classification of fibers is whether or not they occur in nature. Natural fibers include organic fibers produced by animals (keratin), organic fibers produced by grasses, vegetable plants, and trees (cellulose), and inorganic fibers occurring naturally in minerals (asbestos). As shown in Figure 1, the category of natural fibers spans organic and inorganic categories. Cotton, wool, and asbestos are examples of natural fibers from the organic cellulose, organic keratin, and inorganic element mineral categories, respectively. Regenerated cellulose fibers span the categories of natural fibers and synthetic polymer fibers.

Since ancient times, engineers and builders have used natural fibers as reinforcing materials in mortar and bricks. A historical example is the construction of the ziggurat of Dur-Kurigalzu, which was built in Mesopotamia in the 14th Century BC using sun-dried bricks and mortar reinforced with horsehair [9]. Today Natural Fiber-Reinforced Polymer (NFRP) composites are gaining recognition in the field of renewable building materials [10]. Compared with synthetic fibers, natural fibers are abundant, renewable, and inexpensive to produce, although they have relatively low strength and are susceptible to moisture [11].

A simple distinction between natural and synthetic fibers is in their production, i.e., natural fibers are harvested while synthetic fibers are manufactured. Three major subcategories of natural fibers are cellulose fibers, keratin fibers, and asbestos fibers. Cellulose and keratin fibers are discussed below, while asbestos fibers are briefly discussed later as inorganic natural fibers. Regenerated cellulose fibers, which span the natural and synthetic categories, are discussed in the natural category.

#### 2.1.1. Cellulose Fibers

Cellulose is a naturally occurring long-chain polymer of glucose molecules produced by plants and trees. All cellulose fibers are organic; however, they can be naturally occurring or synthetic, as in the case of regenerated cellulose.

##### Vegetable Plant Fibers

Vegetable (or “vegetal”) plant fibers are naturally occurring cellulosic fibers. Cotton is a common example of a vegetable plant cellulose fiber, while many other common examples are shown in Table 2, categorized according to the part of the plant they come from. Plant fibers come from various parts of the plant to include the seed (cotton), stem or bast (flax and hemp), leaf (sisal) and husk (coconut).

##### Seed Fibers

Cotton fibers are 95% to 99% cellulose and are made of long-chain carbohydrate molecules that form a skin, primary wall, winding layer, secondary wall, lumen wall, and lumen [12]. Unlike brittle glass fibers that do not strain prior to breaking, and ductile nylon fibers that strain significantly, cotton fibers exhibit unique “fibrillary” failure under a tensile load within the fiber’s secondary wall, which allows a typical 4% to 8% elongation at break [12]. By comparison, wool fibers elongate by 25% to 40%, while synthetic polyester fibers reach over 50% elongation at break [12].

The harvesting and production of cotton fibers is accomplished through a basic mechanical separation of cotton lint from the seeds with spinning cylindrical drums, combs, brushes, and finely spaced ribs. Eli Whitney invented the cotton gin in 1793 and patented his invention in 1794. Modern day cotton is processed several times through the use of delinting and cleaning machines to remove twigs, short fibers known as linters, and foreign materials using the same concepts as Whitney’s original design.

Cotton has a high degree of polymerization (9000–15,000) compared to other natural fibers including wood pulp (600–1500) and viscose Rayon (250–550) [13]. Cotton fibers come in lengths of 10 to 65 mm, and diameters of 11 to 22 μm. Cotton fibers are not widely used for composite reinforcing, although they have undergone experimental testing for the reinforcement of polypropylene composites [14].

##### Stem (Bast) Fibers

Flax, hemp, jute, and ramie are four common fibers derived from the stems of various plants. The plant stem has three layers: the outer bark, the inner stem core, and a layer in between the bark and core called the bast, phloem, or soft fibers. The phloem translocates soluble organic compounds throughout the plant. Cellulose microfibrils are integrated into elementary fibers, which are bundled together as fiber bundles within the bast of the plant.

Bast fibers are harvested from the plant stems with typical natural fiber processing techniques including rippling, retting, decorticating, breaking, scutching, and hackling. Bast fibers must be separated from the outer woody bark as well as the inner woody core. Rippling is the mechanical removal of leaves, seeds, and other debris from the stem by pulling the stem through iron or wooden teeth. Retting is the process of softening and separating the outer layer of bark from the stem bast core by exposing fiber bundles to water or chemicals. Retting can be accomplished by soaking in water (dam retting or tank retting), exposure to dew (dew retting), or soaking in a solution of caustic soda or sodium carbonate (chemical retting). For stems with tougher outer layers of bark, decorticating is a mechanical technique for removing the outer layer of wooden bark from the stem using rollers and soft hammers that bend and pound the stems. Breaking is the mechanical fragmenting of the woody inner core which is inside the ring of bast fibers, achieved by passing the stems through fluted rollers. Scutching is the mechanical separation of the woody inner core fragments by beating the straw with blunt iron or wood blades. Hackling is the mechanical process of separating the longer linen fibers from shorter fragment fibers by combing the grouping of fibers known as a tow through a bed of pins known as a hackle. All bast fibers are grown, cultivated, harvested, and produced through a various combination of rippling, retting, decorticating, breaking, scutching, hackling, washing, and spinning into filament.

##### Flax Fiber

Flax is a common flowering plant from the Linaceae family harvested throughout the world for linseed oil and flax fibers. Flax fibers are similar to cotton with a crystalline structure which makes them even stronger and more rigid than cotton. Flax fibers are approximately 70 to 75% cellulose, and range in diameter from 12 to 16 μm, with lengths up to 90 cm [15]. Flax fibers have undergone testing as hybrid reinforcing materials for composite materials [16].

##### Hemp Fiber

Hemp is a very fast-growing cannabis plant with a long history of cultivation as a source of fiber. Hemp fibers are approximately 67 to 75% cellulose and are long, strong, and durable; as such, they are used in the production of rope throughout the world. The fibers have diameters ranging from 16 to 50 μm [17] and are under continual study as a hybrid reinforcing compound for composite materials [18].

##### Jute Fiber

Jute fibers come in lengths up to 4 m with diameters from 17 to 20 μm, are approximately 59 to 71% cellulose, and come from the bast tissue of the stem of two species of the Corchorus plant. Nearly all jute fibers are produced in the fertile alluvial regions of India and Bangladesh. Jute fibers are among the strongest vegetable fibers and are commonly used in sackcloth. They have undergone testing as reinforcing compounds for composite polyactide, polypropylene, polyester, and epoxy materials [14,19].

##### Ramie Fiber

Ramie is a flowering plant native to East Asian countries especially China and Korea, but also cultivated for fiber production throughout the world in Japan, India, Malaya, Brazil, Mexico, United States, and Europe [20]. The fibers cannot be extracted from ramie stems by rippling and retting as in flax, hemp, and jute; instead, the woody bark tissue of the stem must be decorticated by heavy pounding or scraping by hand, or with a decorticator machine [20]. After decortication, ramie fibers must be degummed mechanically or chemically [20]. Ramie fibers are exceptionally strong and long, with low elasticity, and have diameters ranging from 25 to 30 μm.

##### Leaf Fibers

Common types of leaf fibers include abaca and sisal.

##### Abaca Fiber

Abaca is a type of banana which is native to the Philippines, where it is primarily harvested for its fibers (also to a lesser extent Ecuador and Costa Rica). Abaca fibers are derived from the leaves of the abaca plant through a labor intensive process of tuxying, stripping, and drying. They have good mechanical strength, buoyancy, long length (up to 3 m), and resistance to decay from salt water, and as such, are used throughout the world as sources for mooring lines and ship ropes.

##### Sisal Fiber

The agave sisalana plant, better known as sisal, is native to southern Mexico. Sisal fibers are extracted from the leaf of the sisalana plant through a process of mechanical deortication. Sisal fibers are coarse and hard, with diameters of 200 to 400 μm, making them unsuitable for textiles or fabrics, but strong and stretchable. Sisal is currently used as a reinforcing fiber in various composite materials [21,22].

##### Husk/Fruit Fibers

Coir is a common husk fiber extracted from the shells of coconuts through a process of dehusking, retting, defibering, and finishing [13]. Like abaca fibers, coir fibers are salt water corrosion-resistant, although they lack the tensile strength and length of the former. Coir fibers have been tested as reinforcing compounds for polypropylene composite materials [23].

##### Wood Fibers

Wood fibers are derived from both hardwood (oak, gum, birch, beech, aspen, eucalyptus) and softwood trees (conifer, pine, spruce, fir, cedar, hemlock, redwood) [13]. In general, hardwood has less lignin content than softwood, and hardwood fibers are short and thick with more impurities. Wood fibers are derived from wood chips that are cooked into a pulp through a “kraft pulping” process which uses sodium hydroxide, sodium sulfide, sulfurous acid, and bisulfite to break down the lignin at 170 °C to create wood pulp [13]. The wood fibers are then bleached, fibrillated, and wet-laid. Softwood fibers typically have a length of approximately 3 mm and diameter of 25 to 30 μm.

##### Regenerated Cellulose Fibers

Regenerated cellulose fibers are produced by extracting the cellulose fibers from vegetable plants and trees through dissolution. Softwood tree pulp and cotton linters are the most common types of cellulose for regeneration into fibers. Viscose is the name of a solution of sodium hydroxide and carbon disulfide that dissolves and removes tree and vegetable plant materials except cellulose fibers. Regenerated fibers are considered both natural and synthetic fibers, since the extracted cellulose is a natural polymer obtained by an artificial method, i.e., by cooking cellulose in viscose. Some examples of regenerated fibers include rayon, acetate, triacetate, modal, polylactic acid, Tencel, and lyocell.

##### Rayon Fibers

Regenerated viscose fibers were invented by Charles Cross and Ernest Bevan in 1891 [13]. The DuPont Fibersilk company began producing viscose in 1921, which was later renamed “rayon,” and the company took on the name DuPont Rayon Company, later becoming the DuPont Textile Fibers Department [13]. Rayon fibers are manufactured fibers composed of regenerated cellulose in which substituents have replaced not more than 15% of the hydrogens of the hydroxyl groups [24]. Lyocell is a generic term for rayon fibers with no substitution of the hydroxyl groups. Modal is a type of rayon fiber derived from reconstituted beech wood cellulose. Tencel is a brand name of both lyocell and modal fibers.

##### Acetate Fibers

Acetate fibers are derived from the treatment of wood pulp, cellulose, or cotton linters with acetic acid [24]. The production of acetate fibers is similar to that of rayon fibers, with the main difference being the use of acetic acid for acetate fibers. The term triacetate is used when more than 92% of the hydroxyl groups are acetylated [24].

#### 2.1.2. Keratin Fibers

Fibers produced naturally by animals are made of keratin, a fibrous structural protein that occurs in hair, feathers, horns, claws, and hooves. Common animal fibers are listed in Table 3.

##### Wool Fibers

Wool is the natural, protective hair-like fiber grown by sheep, rabbits, goats, ox, bison, and similar animals. Wool fibers are obtained through a process of shearing, scouring, and carding. Wool fibers can be electrostatically charged and are very durable, with the ability to flex back and forth more than 20,000 times without breaking, compared to 3000 times for cotton, 2000 times for silk, and 75 times for viscose rayon [13]. Wool is biodegradable, flame resistant, and can absorb 30 times its weight of water and 40 times its weight of oil [13]. Wool fiber diameters range from 15 to 50 μm [25]. Wool comprises multicellular fibers which grow out of skin follicles at a rate of around 100 mm per year [25].

##### Silk Fibers

Silk fibers are the oldest filament of the textile industry, dating back to ancient China. Silk differs from other natural fibers in that it is an extruded protein solution that coagulates on drying, and is not formed from living cells like wool, hair, or cellulose [25]. The most common type of silk is made from the cocoon of the silk moth Bombyx mori [25]. Silk fiber is produced by feeding mulberry leaves to silkworms that extrude it for the construction of cocoons as they enter the larvae stage [25]. Harvested cocoons soaked in hot water can be unrolled to yield approximately 700 m of silk fiber per cocoon [25].

Spiders extrude many different types of silk for various purposes, including catching prey, reproducing, constructing nests, and traveling. Spider silk is not a common material for the textile industry, as it is difficult to produce in large quantities [25].

#### 2.1.3. Comparison of Natural Fibers

Natural fibers are abundant, light weight, and flexible. Natural fibers range in density from 0.14 g/cm^3^ (balsa wood fiber) to 1.54 g/cm^3^ (flax fiber). Spider silk is the strongest known natural fiber, with a tensile strength of approximately 1.4 GPa, i.e., more than twice that of cotton (0.684 GPa) [26,27].

Just as yield strength is the yield force divided by the cross section area, specific strength is the yield force divided by the fiber’s linear density, which is a much more common and easily measured property of textile fibers. Throughout this review, units of specific strength are described in units of megaYuri (MY), which is explored in Annex C. Although the Yuri is not recognized by the international system of units, it is a coherent comparison of official derived SI units of strength (pascal) and density (kg/m^3^) and conveniently describes specific strength in the most basic terms.

Table 4 below compares the density, tensile strength, and specific strength of select natural fibers, ranked from strongest to weakest. A significant characteristic of all natural cellulose and keratin fibers is affinity to water. Cellulose fibers and keratin fibers tend to absorb water and swell.

**Figure 2:**
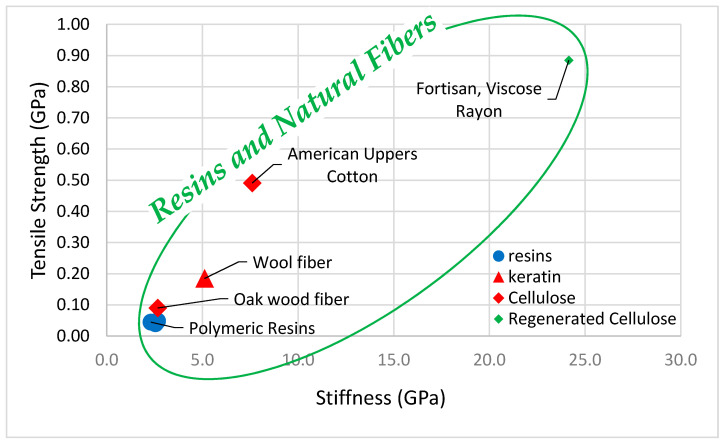
Comparison of Specific Strength and Stiffness of Natural Fibers and Resins.

Figure 2 graphs the tensile strength and tensile stiffness of common polymeric resins. All natural fibers surveyed are stronger and stiffer than the example resin materials.

### 2.2. Synthetic Polymer Fibers

Advancements in polymer sciences have led to the development of high-strength polymer fibers. Fibers are considered synthetic polymers if they are produced from polymers that do not exist in nature. The large number of synthetic polymer fibers creates a challenge for categorization. Table 5 attempts to categorize synthetic polymer fibers into groupings of amides, polyesters, liquid crystalline, olefins, and other common polymers.

#### 2.2.1. Amide Fibers

An amide functional group has the chemical formula -C(=O)-NH-, with a nitrogen atom connected to a carbonyl carbon atom. A polyamide is any polymer linked together by recurring amide functional groups in the polymer backbone. Common amide fibers are nylon 6.6 and nylon 6. Aramid fibers are also amide fibers categorized as liquid crystalline polymer fibers.

Nylon is a generic term for polyamide fibers derived from diamine and dicarboxylic acid [29]. Nylon 6.6 was the very first fully synthetic fiber produced. Wallace Carothers invented nylon 6.6 at the DuPont Research Facility in 1935 [29]. DuPont already had experience in the development of synthetic regenerated cellulose fibers dating back to the DuPont Fibersilk Company in 1925; however, Carothers developed the first noncellulose fully synthetic fiber. Carothers’ invention paved the way for further development of synthetic polymer fibers.

Carothers developed nylon 6.6 by polymerizing hexamethylenediamine (six carbon atoms) and adipic acid (six carbon atoms) into polyhexamethyleneadipamide. Nylon 6, also known as Perlon or PA6, is a polyamide fiber based on only one monomer with six carbon atoms first produced in 1938. There are many other nylon materials and fibers; however, nylon 6.6 and Perlon are the most common. Raw materials used to produce nylon fibers include benzene from coke production and oil refinement, 1,4-butadiene from oil refinement, and furfural from oat hulls or corn cobs [29]. Nylon 6.6 and Perlon are produced through a condensation reactions at approximately 260 °C along the following equations [33].

$$\mathrm{Nylon}:\;n{\mathrm{NH}}_{2}{\left({\mathrm{CH}}_{2}\right)}_{6}{\mathrm{NH}}_{2}+n\mathrm{HOOC}{\left({\mathrm{CH}}_{2}\right)}_{4}\mathrm{COOH}={\left\{\mathrm{NH}{\left({\mathrm{CH}}_{2}\right)}_{6}\mathrm{NHCO}{\left({\mathrm{CH}}_{2}\right)}_{4}\mathrm{CO}\right\}}_{n}+nH_{2}O$$

$$\mathrm{Perlon}:\;{\left\{{\mathrm{NH}}_{2}{\left({\mathrm{CH}}_{2}\right)}_{5}\mathrm{COOH}\right\}}_{m}+{\left\{{\mathrm{NH}}_{2}{\left({\mathrm{CH}}_{2}\right)}_{5}\mathrm{COOH}\right\}}_{n}={\left\{{\mathrm{NH}}_{2}{\left({\mathrm{CH}}_{2}\right)}_{5}\mathrm{COOH}\right\}}_{m+n}+H_{2}O$$

Nylon and Perlon are both thermoplastic polymers, and their fibers are manufactured by melt extrusion through a spinneret and drawing to desired fiber diameter sizes. Perlon has a melting temperature of 215 °C, while nylon 6.6 has a melting temperature of 265 °C [34]. Table 6 shows the density, tensile strength, and specific strength for Perlon and nylon fibers.

#### 2.2.2. Polyester Fibers

Polyester fibers are produced by a chemical reaction involving coal, petroleum, air, and water. French chemist Joseph-Louis Gay-Lussac first synthesized polyester from lactic acid in 1833 [36]. Starting in 1928, Carothers at Dupont synthesized many types of aliphatic polyesters, while E. W. Spanagel synthesized polyethylene terephthalate (PET) at Dupont in 1934 [36]. Later, British chemists John Rex Whinfield and James Tennant Dickson from Imperial Chemical Industries (ICI) were credited with inventing polyester fibers in 1941 [37]. Whinfield and Dickson observed that Carothers at Dupont in the United States had not investigated polyesters formed by ethylene glycol and terephthalic acid [36]. Whinfield and Dickson partnered with British inventors W.K. Birtwhistle and C.G. Ritchie to patent the production of polyester through polymerization of ethylene terephthalate to form PET [37] Dupont purchased the patent rights in 1945 and began producing polyester fibers in the United States in 1950 [37]. Dupont began selling polyester fibers under the tradename Dacron in 1953, while ICI began selling polyester fibers under the tradename Terylene in 1955 [36]. Today polyester fibers are the most widely used synthetic textile fibers on the market [36].

There are many different types of polyester fibers, but they are all produced under a condensation reaction, and all types contain the basic ester functional block, COO [33]. PET fibers are a produced in a reaction of ethylene diglycol and therephthalic acid at 290 °C and 400 kPa with the following equation [33]:

$$n\mathrm{HO}{\left({\mathrm{CH}}_{2}\right)}_{2}\mathrm{OH}+n\mathrm{HOOC}\left(C_{6}H_{4}\right)\mathrm{COOH}=\{\mathrm{OC}\left(C_{6}H_{4}\right)\mathrm{COO}{\left({\mathrm{CH}}_{2}\right)}_{2}O\}n+nH_{2}O$$

Polyester is a thermoplastic polymer that melts at between 245 and 260 °C and can reform into newly made products, making it easy to recycle [33]. Polyester is melt-spun into fibers through a spinneret at a temperature between 265 and 290 °C at rates of up to 4000 m/min [33]. Dupont-Akra Polyester, under DAK Americas, produces polyester fibers today under trade names Dacron and Delcron.

#### 2.2.3. Liquid Crystalline Polymer Fibers

Liquid Crystalline Polymers (LCP) are a category of polymers that demonstrate some degree of crystalline structure as a solid, and disordered amorphous structure as liquid [38]. Thermotropic LCPs form liquid crystals when heated and melted. Lyotropic LCPs are formed by dissolving solids into solution [39] Thermotropic LCPs can be melted and reformed into fibers by melt-spinning and melt extrusion methods while lyotropic LCP fibers require some form of solution or gel spinning. LCP fibers can be separated into three categories: aromatic polyamides (aramids), aromatic heterocycles with lyotropic behavior, and aromatic copolyesters with thermotropic behavior [38].

##### Aramid Fibers

Aromatic polyamide fibers, better known as aramid fibers, are manufactured fibers in which the fiber-forming substance is a long-chain synthetic polyamide with at least 85% of the amide linkages attached directly to two aromatic rings [24]. Whereas nylon fibers connect carbon atoms as the repeating unit between amide groups, aramid fibers connect benzene rings as the repeating unit between amide groups. Aramid fibers are derived from a reaction between an amine group and a carboxylic acid halide group.

Aramid fibers cannot be melt-spun, as they decompose prior to melting. Rather, they are produced by solution spinning techniques including dry jet-wet spinning [38]. Stretching and heat treating as-spun aramid fibers at temperatures of 150 to 550 °C for short periods of time have been shown to increase the crystallization orientation and enhance the fiber mechanical properties [38]. Two categories of aramid fibers are para-aramid and meta-aramid.

##### Para-Aramid Fibers

Examples of para-aramid polymers include PBA, PPTA, and PBIA [38]. DuPont synthesized the first para-aramid fiber, known as Kevlar, from PPTA in 1965. Stephanie Kwolek from DuPont is credited with inventing Kevlar. Kevlar fibers are synthesized in condensation reactions from para-phenylenediamine and terephthaloyl chloride. Twaron is a para-aramid fiber similar to Kevlar first produced by Akzo in 1978, and now produced by Teijin Aramid of Japan [38].

##### Meta-Aramid Fibers

MPIA is an example of a meta-aramid polymer [38]. DuPont produced the first meta-aramid fiber based on MPIA known as Nomex in the early 1960s. Wilfred Sweeny, a scientist at DuPont, is credited with the discoveries leading to Nomex. Nomex fibers are synthesized through a condensation reaction from meta-phenylenediamine and isophtaloyl chloride. Nomex fibers have a lesser degree of crystallinity than Kevlar fibers [40]. Nomex 430 is considered highly crystalline and is produced from 100% Nomex [41]. Continuous exposure of Nomex fibers to temperatures of 200 °C or higher reduces the degree of crystallinity to zero [42]. Teijinconex is another MPIA-based meta-aramid polymer fiber made by Teijin Aramid of Japan [38].

##### Aromatic Heterocycle Liquid Crystalline Fibers

Heterocyclic polymers with lyotropic behavior have fully aromatic molecular structure with fused heterocyclic rings along the main chains [38]. Heterocycle LC fibers can be classified into three categories: polybenzazole (PBO, PBZT, and APBO), polybenzimidazole (PBI), and polypyridobisimidazole (PIPD) [38].

##### PBO Fibers

Polyphenylene Benzobisoxazole (PBO) is a heat-resistant thermoset liquid crystalline polymer with a benzene-fused oxazole ring structure which gives it high strength and low density, first produced by the U.S. Air Force in the 1960s [38]. Zylon is a brand name of PBO fibers produced by Toyobo of Japan. Zylon fibers show very high tensile strength of 5.8 GPa and density of 1.56 g/cm^3^, for specific strength of 3.7 MY. According to Toyobo, Zylon has a melting temperature of 650 °C so it can withstand higher temperatures than comparable strength UHMWPE fibers which decompose between 130 and 136 °C.

##### PBI Fibers

Polybenzimidazole (PBI) thermoset liquid crystalline fibers were developed by Celanese Corporation in 1983 and are still produced by as Celazole PBI Performance Products [38]. PBI fibers, similar to all aromatic structure fibers, demonstrate thermal and chemical stability [38].

##### PIPD Fibers

Polydiimidazo pyridinylene dihydroxy phenylene (PIPD) fibers were developed by Sikkema and colleagues at Akzo Nobel under the trade name M5 in the 1990′s and are currently produced by Magellan, which is now a division of Dupont [38]. PIPD fibers are noted for their bidirectional network of hydrogen bonds in the molecular chain, formed by the hydroxyl groups on the phenyl ring [43]. M5 fibers in particular are noted for their exceptional compressive strength, the highest of all polymeric fibers, at 1.7 GPa [43,44,45].

##### Copolyester Liquid Crystalline Fibers

The third type of liquid crystalline fiber is known as thermotropic aromatic copolyester fibers [38]. These fibers are unique, in that they maintain their molecular structure at high temperatures, which enables the application of a melt spinning process [38]. The most common copolyester fiber is Vectran made by Kuraray of Japan [38]. Vectran is manufactured into a high performance fiber from p-hydroxybenzoic acid and 6-hydroxy-2-naphthoic acid (HBA/HNA) [38]. Table 7 shows the density, tensile strength, and specific strength for liquid crystalline synthetic fibers.

#### 2.2.4. Olefin Fibers

Olefin, also known as alkene, is an unsaturated hydrocarbon material made up of compounds of carbon and hydrogen with one or more double or triple bond. Two common types of olefin polymers used to make fibers are PolyEthylene (PE), with chemical formula (C_2_H_4_)n, and PolyPropylene (PP), with chemical formula (C_3_H_6_)n.

##### Polyethylene Fibers

Polyethylene fibers include High Density Polyethylene (HDPE) and Low Density Polyethylene (LDPE). HDPE is produced through extrusion at low pressure and low temperature, while LDPE is produced through high pressure and high temperature. Polyethylene fibers have high strength and high modulus with low density resulting in high tenacity fibers.

Ultra High Molecular Weight Polyethylene (UHMWPE) fibers are HDPE fibers with extremely high molecular weight. The degree of polymerization of UHMWPE fibers is well over one million. UHMWPE fibers are manufactured through a dry gel-spinning process described by U.S. Patent 5068.073 by Pennings et al. dated 26 November 1991 [52]. UHMWPE is heated between 180 and 250 °C and extruded into a spinneret, from which UHMWPE fibers are drawn through spinning nozzles at a rate of 500 to 4000 m/min [52]. Common brand names of UHMWPE fibers are Dyneema, Tsunooga, IZANAS, and Spectra.

Dyneema, Spectra, and Tsunooga are UHMWPE fibers produced by DSM, Honeywell, and Toyobo respectively, all with a density of 0.97 g/cm^3^. DSM reports a tensile strength of 4.1 GPa for Dyneema SK99 resulting in specific strength of 4.2 MY, which is higher than the specific strength of the strongest carbon fibers [53]. Honeywell reports a tensile strength of 3.7 GPa for Spectra 1000 while Toyobo Tsunooga shows 1.4 GPa, resulting in specific strengths of 3.8 MY and 1.43 MY respectively. DSM Dyneema fibers are manufactured with diameters between 12 and 21 μm, while Honeywell Spectra come in slightly larger sizes, i.e., 17 to 41 μm [54]. UHMWPE fibers are resistant to water, chemicals, and UV light.

A significant drawback for UHMWPE fibers is their poor thermal performance. The strength of UHMWPE fibers is based on a very large number of weak Van der Waals forces connecting extremely long parallel linear molecular chains. Although demonstrating melting temperatures between 126 and 145 °C, UHMWPE fibers lose strength at much lower temperatures, i.e., between 70 and 80 °C, as Van der Waals forces break down between linear molecular chains. UHMWPE fibers do not become brittle until they reach temperatures below −150 °C.

##### Polypropylene Fibers

Polypropylene is also a polymer of the olefin family with chemical formula (C_3_H_6_)n. Polypropylene fibers are manufactured and commonly employed as nonwoven HEPA-quality air filter units for home and office [55]. Polypropylene fibers are hydrophobic, water repellent, not easily dissolved, and washable [56]. The most common method of producing polypropylene fibers is by melt-spinning, where polypropylene granules are melted at temperatures around 210 to 270 °C, extruded and drawn through a spinning die, and quenched at about 30 °C [56]. Polypropylene fibers have a melting temperature of 165 °C; however, polypropylene molecular chains degrade at temperatures above 100 °C, which limits their use in higher temperature applications. Polypropylene fibers are significantly weaker than UHMWPE fibers, at only 0.6 GPa tensile strength. With a density of 0.91 g/cm^3^, polypropylene fibers have a specific strength of around 0.65 MY.

Polypropylene fibers are useful components in boat mooring ropes because of their ability to float, and their hydrophobicity [56]. However, polypropylene fibers deteriorate over time through an aging process, i.e., oxidation in air. Polypropylene fibers also deteriorate through exposure to UV light [56] and become brittle and rapidly lose strength at temperatures below 0 °C and when they age [56]. Table 8 shows the density, tensile strength, and specific strength for selected olefin synthetic fibers.

#### 2.2.5. Acrylic Fibers

Acrylic fibers are manufactured fibers in which the fiber-forming substance is any long-chain synthetic polymer composed of at least 85% by weight acrylonitrile monomer units [24]. If the fiber is composed of less than 85% but greater than 35% acrylonitrile, the fiber is called “modacrylic” [24].

Polyacrylonitrile (PAN) fibers were first produced in 1938, and DuPont began producing commercially available PAN fibers under the name Orlon in 1944 [59]. PAN has nitrile functional groups (CN) attached to a polyethylene backbone with a linear formula (C3H3N)n. The Sohio process combines propylene, ammonia, and oxygen through a heterogeneous vapor-phase catalytic reaction called propylene ammoxidation at 400 to 510 °C and 50 to 200 kPa pressure which produces acrylonitrile, acetonitrile, hydrogen cyanide, carbon monoxide. Acrylonitrile is then polymerized into PAN in a solvent, typically dimethlformamide (DMF), dimethylacetamide (DMAc), dimethylsulfoxide (DMSO), propylene carbonate, or aqueous sodium thiocynate at 45 to 55% solution [59].

PAN fibers are produced through dry-spinning, wet-spinning, or electro-spinning. Wet spinning involves replacing the solvent with a nonsolvent liquid in a diffusion process. The polymer solution is extruded through a spinneret with between 2000 and 360,000 holes that are each 0.05 to 0.38 mm in diameter, into a nonsolvent solution. The fibers are drawn in a tow, stretched at a 2× ratio by the first roller, washed and stretched again in hot water or steam, and then dried. Dry spinning involves extruding the polymer solution at 90 to 140 °C through a stainless-steel spinneret with 2800 holes 0.1 to 0.3 mm diameter. The resulting tow of fibers is subjected to a heated airflow for the evaporation of the solvent and stretched at a ratio of 2 to 10 depending on the desired properties [59]. A combined dry-wet spinning process involves extruding PAN fibers from a dry spinneret with a short air gap prior to entering the solution bath. There have also been attempts at melt-spinning PAN; however, the challenge is polymer degradation prior to reaching its melting point of 320 °C [59].

PAN fibers can be produced by electrospinning, either through a syringe needle or needle-less setup. The advantage of electrospinning is the ability to achieve submicron nanometer sized fiber diameters. Conventional dry-spinning and wet-spinning of PAN fibers results in fiber diameters of 5 to 500 μm, while electrospinning results in fibers in the 5 to 500 nm range [59]. Stretching (drawing) PAN fibers during the spinning process before solidification serves to reduce the fiber diameter while aligning the polymer molecules, which increases the resulting fiber tensile strength [60]. PAN fibers are noncombustible, flame resistant, corrosion resistant, and mildew and mold resistant. PAN does not degrade from UV light, and does not easily dissolve in solvents. PAN fibers decompose in air at temperatures above 327 °C.

**Figure 3:**
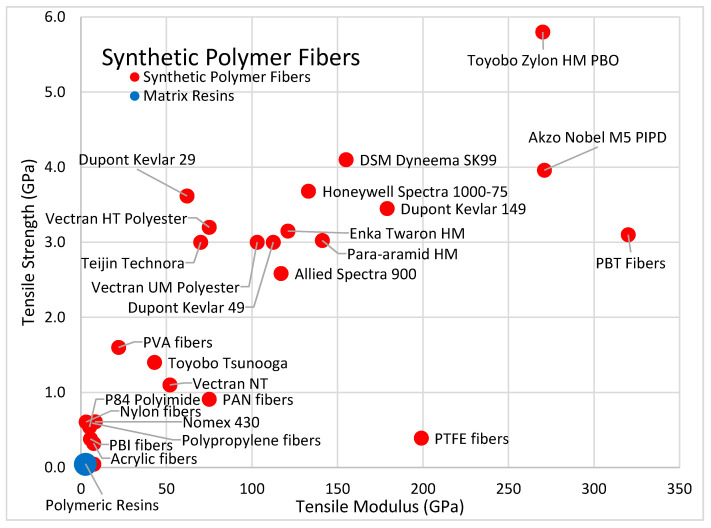
Strength and modulus comparison of synthetic polymer fibers.

Figure 3 compares the strength and modulus of select synthetic polymer fibers with the strength and modulus of typical polymeric matrix resins.

### 2.3. Carbon Fibers

Carbon fibers are already common as reinforcements in additive manufacturing, both as short-cut carbon fibers mixed in the matrix material and as long continuous fibers placed by design. Carbon fibers are produced either by Chemical Vapor Deposition (CVD) or by carbonizing precursor polymer fibers, and can be categorized by their precursor, as PAN-based, pitch-based, viscose-based, or lignin-based. Pitch and PAN are petroleum synthetic byproducts, while viscose rayon (regenerated cellulose) and lignin are natural polymers. Currently PAN-based carbon fibers hold approximately 96% of the market share, while pitch-based carbon fibers account for the remainder [61]. Lignin is an abundant natural resource that has gained recent attention and interest [62].

Thomas Edison may have been the first to use carbon fibers in 1879 with the invention of the incandescent lightbulb, for which he carbonized cotton threads and bamboo slivers as filaments [63]. Roger Bacon is credited with discovery of CVD carbon fibers in 1956 at the Union Carbide Parma Technical Center near Cleveland OH, as he was attempting to find the triple point of carbon with high temperature and pressure. Bacon noticed that at lower pressures, stalagmite-like carbon deposits formed on the lower electrode of his equipment. As he broke open a deposit, he found a bundle of carbon whiskers less than 10 µm in diameter [63]. Three years later in 1959 Akio Shindo in Japan produced the first carbon fibers by thermally stabilizing and carbonizing PAN precursor fibers [60]. Leonard Singer later developed the first pitch-based carbon fiber by applying stress and heat to a pitch precursor filament, obtaining a carbon fiber with unusually high tensile modulus of nearly 1000 GPa [60].

Carbon fibers are manufactured with a wide variety of sizes, strengths, and moduli, depending on how they are produced. Carbon fibers are classified in five grades: ultrahigh modulus (UHM), high modulus (HM), intermediate modulus (IM), standard modulus (SM), and high strength-high strain (HT). PAN-based IM carbon fibers have the highest tensile strength, while the pitched based UHM and HM carbon fibers have the highest tensile modulus.

The Toray T1100G reaches a tensile strength over 7 GPa. With a density of 1.79 g/cm^3^, the Toray T1100G has a specific strength of nearly 4 MY. Based on production technique, PAN based carbon fibers can also achieve a high modulus. The Toray M46J carbon fiber has a tensile modulus of 436 GPa, while the Toray M60J carbon fiber has a tensile modulus of 588 GPa. The increase in stiffness comes at the loss of strength, as the M46J and M60J fibers have specific tensile strengths of 2.3 and 2.0 MY. PAN-based carbon fibers were first commercialized in 1950 by Dupont under the trade name Orlon [60].

Carbon fibers from pitch have lower strength than carbon fibers from PAN, but the pitch-based UHM and HM fibers have a higher stiffness modulus. The Thornel K-1100X pitch-based carbon fiber has a tensile modulus of 999 GPa. Both isotropic and mesophase pitch are precursors for pitch-based carbon fibers, produced through melt-spinning. After fibers are formed, they are stabilized in air at 275 to 350 °C, and then carbonized at 1400 °C. The fibers become graphite if heated to 2500 °C in nitrogen [60].

Table 9 shows the density, tensile strength, and specific strength for selected PAN-based and mesophase pitch-based carbon fibers.

For PAN- and pitch-based carbon fibers alike, the carbonization/graphitization temperature and duration are important parameters, determining the thermal and electrical conductivity. Higher temperatures during the carbonization process serve to reduce point defects and increase crystalline perfection and orientation [60]. The improved crystalline orientation along the fiber axis increases the tensile modulus, thermal conductivity, and electrical conductivity [60]. The thermal conductivities of PAN- and pitch-based carbon fibers range from 5 to 156 W/(m⋅K) and 26 to 800 W/(m⋅K) respectively.

Viscose-based carbon fibers, first developed in the 1950s, are synthesized through the kraft pulping process described in Section 2.1.1 for wood fibers and regenerated cellulose fibers [62]. Softwood and cotton linters are the most common sources of cellulose in viscose-based carbon fibers. Lignin is an abundant renewable source of natural carbon polymer derived from within vegetable plant cells along with cellulose and hemicellulose [62]. Lignin is an amorphous thermoplastic aromatic biopolymer with 40 to 50% carbon content, as compared to the approximately 30% carbon content of viscose rayon [62]. The high carbon yield, abundance, and sustainable nature of lignin has attracted recent interest as an alternative carbon fiber precursor to PAN. However, currently, neither viscose-based nor lignin-based carbon fibers come close to achieving the tensile strength and modulus of PAN-based and pitch-based carbon fibers.

**Figure 4:**
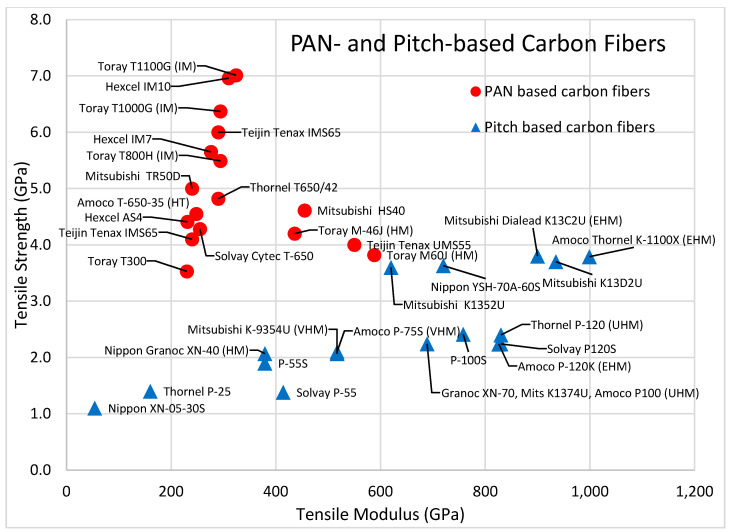
Strength and Modulus Comparison of Carbon Fiber.

Carbon fibers are popular in composite materials based on their mechanical characteristics. Carbon fibers maintain integrity and withstand high temperatures without losing strength. Figure 4 shows the difference in tensile strength and tensile stiffness between PAN-based and pitch-based carbon fibers. PAN-based carbon fibers generally have higher tensile strength, while pitch-based carbon fibers have higher tensile stiffness.

### 2.4. Ceramic Fibers

Ceramics are inorganic, nonmetallic, nonplastic, nonglass compounds. Ceramic fibers are distinguished from other inorganic fibers in that they are not produced by solidifying melts [70]. Oxides, nitrides, carbides, and borides are examples of ceramic materials. Ceramic fibers withstand high temperature conditions. In contrast to carbon and boron fibers, ceramic fibers do not oxidize at moderate or high temperatures [70].

**Figure 5:**
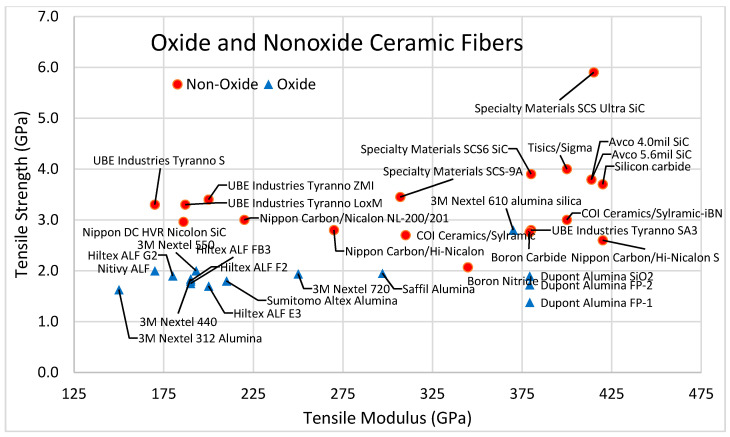
Strength and Modulus Comparison of Ceramic Fibers.

Ceramic fibers are categorized into two categories according to their composition: oxides (aluminum oxide, zirconium oxide, mullite) and nonoxides (silicon carbide, silicon nitride, alumina silicate). The tensile strength of oxide fibers ranges from 1.7 to 3.1 GPa, with a tensile modulus between 150 and 380 GPa, while nonoxide ceramic fibers generally have higher tensile strength, i.e., between 3.4 and 5.9 GPa, and a tensile modulus between 317 and 415 GPa. A comparison of tensile strength and tensile modulus for various oxide and nonoxide ceramic fibers is shown in Figure 5.

#### 2.4.1. Nonoxide Ceramic Fibers

Most nonoxide ceramic fibers are silicon carbide, while some include titanium, zirconium, or other elements to obtain various properties [70]. Three major producers of silicon carbide fibers include Nippon, UBE, and Specialty Materials. Specialty Materials uses a CVD process similar to CVD-based carbon fibers. A mixture of alkane and chlorosilane condenses onto a tungsten or carbon core, resulting in a column grain structure with growth perpendicular to the tungsten or carbon core [70]. The Nippon company produces Nicalon fibers through a continuous yarn process, invented by S. Yajima in the 1970s, involving the conversion of dimethyldichlorosilane into polycarbosilane, heating in argon at 470 °C for 8 to 14 h, then melt-spinning [70]. The UBE “Tyranno” brand of carbide fiber are produced in a similar manner, but also contain zirconium, aluminum, and titanium, which give the fiber additional strength [70]. There were three major generations of silicon carbide fibers, which have resulted in a significant increase in the tensile modulus, in the case of Nippon Nicalon, from 200 to 400 GPa [71]. Appendix D compares the composition of nonoxide ceramic fibers. A comparison of nonoxide ceramic fibers is given in Table 10.

#### 2.4.2. Oxide Ceramic Fibers

Most oxide ceramic fibers comprise alumina or alumina-silica, with alumina contents of up to 72% [75]. Although not as strong as nonoxide ceramic fibers, continuous oxide ceramic fibers demonstrate good strength, chemical stability, a high modulus, and a high melting point which enables their use in applications up to around 1200 °C [75]. Oxide ceramic fibers tend to creep under high temperature loads [70]. High alumina fibers are made from water-soluble or sol-gel precursor products, or from an organometallic precursor, as in the case of Sumitomo’s Altex Alumina fiber [70].

Oxide ceramic fibers are produced by a sol-gel process, which is a method of producing high temperature-capable ceramic fibers with a low temperature process. Small sized metal alkoxide nanoparticles are mixed in a solvent. Particle grain sizes less than 100 nm are preferred [75]. The molecules connect together in a liquid colloidal suspension and form polymers in the form of a gel. The gel can then be formed into fibers through dry-spinning or electrospinning. The sol-gel process can produce single-component oxide ceramic fibers, or multicomponent oxide ceramic fibers, and can be used to produce ceramic oxides as well as glass [76]. After spinning the salt solution, the resulting fibers are heat treated to remove the volatile components of the sol that enabled fiber formation [75]. Nextel, Hiltex, Cerafib, and Nivity producers of continuous oxide ceramic fibers [75].

Saffil is a trade name for short-cut alumina fibers made by Unifrax, primarily used in insulation applications up to 1600 °C. Saffil fibers are 96% aluminum oxide and 4% silica, and have a density of 3.3 g/cm^3^, tensile strength of 2.0 GPa, and melting point of 2000 °C. Ceramic oxide fibers are shown in Table 11. Unifrax also produces Fibermax short-cut oxide ceramic fibers. Additional short-cut alumina fibers in bulk are produced by Misubishi Plastic (Maftec), Ibiden (Ibiwool), Rath (Altra), and Morgan Thermal Ceramics (Alphawool) [75].

### 2.5. Oxide Fibers: Quartz and Glass

Quartz and glass are oxides in addition to the oxide ceramics described above, based on silica compounds. Quartz is classified as both a ceramic oxide and a silicate mineral made from nearly 100% silica (SiO_2_). Quartz is the second most abundant mineral in the earth’s crust, at approximately 12.5%.

Quartz is distinguished from glass because of its crystalline structure. Silica has a melting temperature around 1710 °C. If silica is heated above 1200 °C and then cooled slowly in ambient conditions, it forms a crystalline structure and becomes quartz; if it is heated and then cooled rapidly, however, it retains an amorphous, noncrystalline molecular structure and forms glass. Quartz is considered a ceramic because of its crystalline nature, while glass is not considered a ceramic, because of its amorphous nature.

Quartz and glass fibers are produced with a five-step method of continuous filament melt drawing, comprising batching, melting, fiberizing, coating, and drying. [79]. Batching is the process of cleaning, weighing, combining, and mixing component materials. Bulk materials are mixed together in a batch. The melting process occurs at high temperatures (approximately 1400 °C for glass) as the materials are melted and mixed. Some production techniques involve the intermediate step of forming glass marbles that are cooled, inspected, and transported to another facility for remelting and forming into fibers. Glass fiberization involves extrusion and attenuation. Molten glass is extruded through heated bushing plates with very fine orifices. The bushing plates, constructed from platinum and rhodium, have between 400 and 8000 holes approximately 1 mm in diameter [80]. The extruded glass fibers are then attenuated. Attenuation is the mechanical process of drawing and stretching the fibers into longer lengths with smaller diameters, which is normally accomplished by winding the fibers onto a collet wheel that is rotating at a rate adequate to draw and stretch the fibers [79]. Coating is the step of applying a chemical binding agent to the surface of the fiber known as sizing. The sizing is added typically at 0.5 to 2.0% by weight of the fiber, helps lubricate and protect the fiber. The binding agent is important for the resultant interfacial shear strength of the fiber and composite resin. The final step in glass and quartz fiber production is drying and packaging. Continuous filament fibers formed in this manner are known as textile fibers, collected as a roving, and can be woven into fabric or applied as reinforcing compounds in additive manufacturing [80].

#### 2.5.1. Quartz Fiber

Quartz fibers have a crystalline molecular structure obtained by slowly cooling fibers of molten, nearly pure silica. Compared with glass fibers, quartz fibers are much stronger and lighter. Saint-Gobain is a company that specializes in the production of quartz fibers called Quartzel, which show a tensile strength of 6.0 GPa and a density of 2.20 g/cm^3^. Quartz fibers can be used as long continuous fibers or chopped into shorter segment lengths. Quartz fibers do not melt until the temperature exceeds 1650 °C [81], demonstrate chemical stability, and are not affected by halogens or acids, except hydrofluoric acid, hot phosphoric acid, and alkalies [81]. Furthermore, they have high electrical resistivity [81]. The composition and strength of quartz fibers are noted and shown in Appendix D.

#### 2.5.2. Glass Fiber

Although the history of glass fiber extends back to 1600 B.C. Egypt, physicist Ferchault De Reamur first made glass fibers into a textile yarn in 1713 [80]. Glass fibers were first produced for air filtration purposes about 100 years ago, as a secondary product of a glass bottle manufacturing company in Newark Ohio [79]. In 1935, Owens Corning began producing glass fiber wool for thermal insulation from the same glass bottle facility. Today, glass fibers are the most common fiber material on the market for composite reinforcement as well as air filtration, thermal insulation, and optical cables. Glass fibers are inexpensive, simple, and plentiful.

Glass fibers are produced by melt drawing, as described above, as well as by centrifugal melt spinning. In centrifugal melt spinning, molten glass is poured into a heated metal bin that spins over 2000 rpm and is maintained at temperatures over 1000 °C. The centrifugal force extrudes glass fibers through thousands of orifices in the sides of the bin. The molten glass hardens as it cools in the air and forms glass fibers on the collector.

Glass fibers are synthetic vitreous silicate fibers, containing somewhere between 55% and 75% silica [79]. Elements from the first two columns of the periodic table are typically present as cations, known as network modifiers to the glass, while oxides of various elements are known as alternative network formers, including alumina, boron trioxide, zirconia, magnesia, calcia, zinc oxide, titania, sodium oxide, potassium oxide, lithium oxide, and iron oxide [79]. Different compositions of these materials with silica give glass fibers very different properties, giving rise to many categories of glass fibers, including Alkali (A), All Fiber (AF), Alkali Resistant (AR), Chemical resistant (C), basalt-glass, low Electrical (E) conductive, high strength (S and R), and low Dielectric (D) [79]. Appendix C provides a comparison of material composition of glass, quartz, and basalt fibers.

A-glass is an alkali-lime bottle-glass, so-named because of its high alkali content. A-glass is made with silica, calcia, and sodium oxide. A-glass fibers have the highest concentration of silica, i.e., approximately 71% [79]. AF-glass contains boron trioxide and is a common material for windowpanes. AR-glass fibers have a high concentration of zirconium oxide, resist corrosion in high alkaline environments, and have common use as concrete reinforcement fiber. The original E-glass was an alumino-borosilicate glass with similar composition as A-glass with high concentrations of alumina, boron trioxide, and calcia [79]. E-glass was originally intended for electrical applications, given its low electrical conductivity. E-glasses have high strength, stiffness, electrical resistivity, and chemical durability to resist corrosive behavior of chloride ions in particular [79]. In recent years, E-glass without boron trioxide has gained popularity thanks to its chemical resistance and durability in acid solutions. The higher required temperatures make ECR-glass fibers more difficult to produce but less expensive without typical boron-containing E-glass. E-glass fibers are common reinforcing compounds in plastic composite materials. C-glass is an alkali-lime glass rich in boron trioxide, originally named for chemical applications, which exhibits good chemical resistance [80]. S-glass and R-glass make up the family of high strength aluminosilicate glass fibers, rich in silica, magnesia, and alumina, with tensile strengths 50% greater than E-glass [79]. The specific strength of S-2 glass fiber (2.0 MY) is twice that of C-glass fiber (1.0 MY). D-glass is a borosilicate glass, originally named for its low dielectric constant [80]. M-glass is named on account of its high tensile modulus, while Te-glass is named for its high temperature applications. Table 12 compares the density, tensile strength, and stiffness of glass and quartz fibers, from the strongest to the weakest.

**Figure 6:**
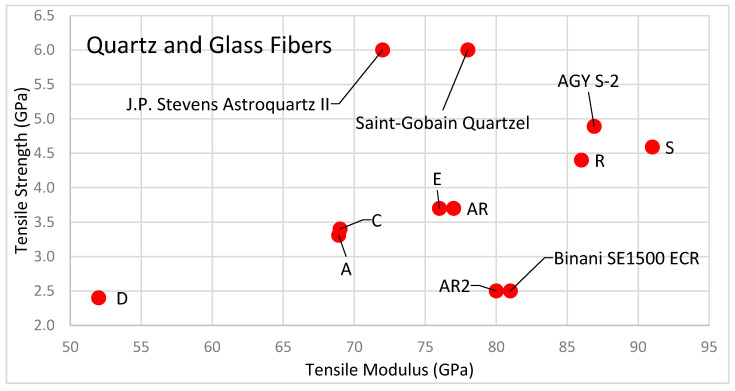
Strength and Modulus Comparison of Glass and Quartz Fibers.

Although well below the strength of carbon fibers, glass fibers have tensile strength much greater than the resins, which improves the resulting composite strength. A comparison of tensile strength and tensile modulus of quartz and glass fibers are shown in the Figure 6.

### 2.6. Mineral Fibers

Mineral fibers are natural inorganic fibers including quartz, asbestos, boron, and mineral wools. Basalt fibers are sometimes described as mineral fibers; however, basalt is volcanic rock, and not a mineral.

#### 2.6.1. Asbestos Fibers

Asbestos is a naturally-occurring, long thin fibrous material formed on silicate minerals. Asbestos fibers are divided into two classifications, as shown in Table 13: serpentine (curly fibers) and amphibole (needle-like fibers). Chrysotile is the only form of serpentine asbestos, while amphibole asbestos includes amosite, crocidolite, tremolite, anthophyllite, and actinolite. Chrysotile is the most common form of asbestos found in buildings constructed before 1980. Asbestos fibers are highly heat resistant and make excellent thermal and electrical insulation materials. In the 1970s, asbestos-related health hazards became known, which significantly limited their use in construction. Airborne asbestos fiber particles become lodged deep into lungs and cause cancer. Although not banned in the United States, the use of asbestos is illegal in more than 50 countries throughout the world, including the UK, Australia, and Canada [86].

#### 2.6.2. Boron Fibers

Boron fibers were first produced in 1959 by Talley at Texaco Experiment Incorporated under the direction of the US Air Force Materials Laboratory [87]. Boron fibers are typically produced through a CVD process in a tube reactor onto either a tungsten or carbon core at 1300 °C. Boron trichloride gas and hydrogen pass through the chamber and deposit onto a tungsten wire that is approximately 12 μm diameter. The halide reduction chemical reaction converts only approximately 10% of boron trichloride gas into boron and emits hydrogen chloride gas. Boron fibers are lightweight, strong, and brittle. Boron fibers have potential in space applications for their radiation shielding characteristics [87].

### 2.7. Volcanic Rock: Basalt Fibers

Basalt rock comes from volcanic magma flow, which covers approximately 70% of the earth’s surface. Frenchman Paul Dhe was the first to successfully extrude fibers from molten basalt in 1923, receiving a U.S. patent for their production [88]. Basalt fibers are produced directly from molten basalt rock at between 1400 and 1600 °C with no other additives; however, their composition depends entirely on the iron oxide content of the particular basalt material. If molten basalt is quickly quenched, it forms an amorphous material similar to glass; however, if it is allowed to cool slowly, it forms a crystalized pattern similar to that of quartz.

Basalt fibers are unique in that they can be recycled by melting at 1400 °C and reformed into new basalt fibers with the same properties as before. Basalt fibers are thus environmentally friendly and considered a sustainable material, since basalts are the most common minerals on the surface of the earth, and there are no additives or solvents used in their production. Basalt fibers are well known for their mechanical strength and durability, as well as thermal, chemical, electrical, and acoustic insulation properties [88]. Basalt can be formed into fibers known as “mineral wool”, which has a history of use as thermal insulation [79]. Basalt fibers have no reactions with chemicals or water, are noncombustible, do not corrode, and pose no threat to human beings.

Basalt is generally composed of 43% to 47% SiO_2_, 11% to 13% Al_2_O_3_, 10% to 12% CaO, 8% to 11% MgO, and about 15% other oxides. It is ideal for producing fibers containing olivine (2MgFe.O.SiO_2_) and nepheline Na_2_O.Al_2_O_3_.2SiO_2_) [88]. The production of basalt fiber is similar to that of glass fibers, although at a lower temperature and with no additives. Basalt fiber production is simpler than glass fiber production, since the raw material does not have to be protected from the weather. Basalt fibers may be divided into two primary categories: discrete/short fiber such as mineral insulating wool (BF), and basalt continuous fiber (BCF). Mineral wool BF is produced by simple centrifugal melt-blowing of crushed basalt stones, known as the Junkers method, which produces fibers 6 to 10 cm in length and 6 to 10 μm diameter. BCF is produced by melt-drawing through bushing units similar to glass fibers.

Basalt fibers have unique thermal and heat resistance characteristics [88]. When exposed to fire, basalt fibers are nonflammable, do not drip, and do not produce smoke. Exposure to higher temperatures promotes crystallization and a loss of mechanical characteristics. The ability of basalt fibers to resist moisture is a significant difference with glass fibers, which tend to absorb moisture in high humidity conditions. Basalt fibers have excellent chemical corrosion resistance, especially to acids, exceeding the performance of E-glass fibers in both acids and alkalis [88].

### 2.8. Metal Fibers

Although metal fibers have been around for centuries, modern metal fibers were first produced in the 1940s [89]. The Dobeckmum Company is credited with the creating the first metallic fiber used in textile yarn in 1946 [90]. A wide variety of metal fibers are produced from alloys with a diameters ranging from 1 to 80 μm including stainless steel, nickel, nickel alloys, titanium, copper, and other high temperature resistant alloys [13]. Metal fibers take on the mechanical, chemical, and thermal characteristics of the metal itself, including density, electrical conductivity, heat conduction, thermal resistance, and corrosion resistance. Metal fibers are currently used in construction and textile industries in the form of woven mesh, sintered mesh, filament yarns, long continuous reinforcement, and short chopped rovings [30]. Various uses for metal fibers include antistatic applications, filtration media, sound absorption, thermal insulation, flame arresting, medical applications, radar camouflage, microwave detection, high-voltage protection, and capillary use [91]. Metal fibers can be easily formed into shapes with high structural integrity, and work well in high temperature, high pressure, and high corrosive conditions. As such, they are commonly used to reinforce concrete mixes.

Metal fibers are produced by a number of techniques including bundle drawing, foil shaving, machining, melt spinning, and chemical vapor deposition [91]. The ductility of metal fibers enables effective bundle drawing techniques, pressing thousands of metal wires through a die to reduce the diameter of all fibers in the bundle. Bundle drawing presses the fibers against one another, resulting in octagonal cross sections. Recent developments have enabled bundle drawn metal fibers to achieve nano-scale diameters of 200 nm and below [89].

## 3. Mechanical, Thermal, and Chemical Considerations for Fiber Selection

### 3.1. Anisotropic and Isotropic Characteristics

Isotropic is a material property that indicates the same numerical value in all directions, while anisotropic is defined as having differing numerical values for a material property in differing directions. Fibers with amorphous structure, including glass, ceramic, and metallic fibers used as reinforcing compounds in additive manufacturing, are generally isotropic. Carbon fibers are anisotropic, as the basic stacking and folding of turbostratic basal planes creates a significant difference in the strength of carbon fibers in the axial and radial directions. This basic molecular level organized structure creates anisotropic properties, for example, the thermal expansion of carbon fibers is negative in the axial direction but slightly positive in the radial direction. Polymeric fibers are anisotropic, based on their molecular level structures and macromolecular alignment in the axial direction. Cellulose and keratin fibers are naturally anisotropic as well. Table 14 separates fiber types into isotropic and anisotropic categories.

Whether or not the fibers themselves are isotropic or anisotropic, the resulting printed materials from FDM are anisotropic based on short-cut fiber alignment with the printing direction during polymeric composite production. The high degree of fiber alignment creates an anisotropic nature of the printed material to include mechanical, thermal, and electrical properties. Strength, elasticity, stiffness, and thermal conductivity of printed components varies greatly in the axial and transverse directions of printed materials.

### 3.2. Mechanical Considerations

Designers of printed materials must consider the density, strength, stiffness, and rigidity of fibers they are adding into the matrix resin to understand how the characteristics of the composite material will differ from the resin alone. Previous studies have shown that the addition of short-cut carbon fibers into matrix thermoplastics can improve the mechanical properties of printed materials up to a certain point [92]. The addition of carbon fibers tends to increase the printed material tensile strength and tensile stiffness while decreasing toughness, yield strength, and ductility [92]. Studies have shown similar results with the addition of short-cut basalt fibers into ABS materials [93].

The ultimate fiber for use in composite materials would have high tensile strength, compressive strength, low density, high tensile and compressive modulus, high rigidity, high toughness, and good interfacial bonding with the matrix [44]. These are primary mechanical considerations for designers.

#### 3.2.1. Density

The weight and buoyancy of printed composite materials depends on the density and amount of resin and fibers. A comparison of resin and fiber density is presented below. Typical resins used in FRAM have density between 1.0 and 1.4 g/cm^3^. Most fibers used in FRAM are denser than the resin, which decreases buoyancy and increases weight of printed composite materials. In particular, metal fibers are the densest with values ranging upward to 9.0 g/cm^3^, so a high percentage of metal fibers significantly adds mass and weight to the printed composite. Conversely, wood fibers are the least dense so a high percentage of wood fibers will reduce weight and increase the buoyancy of printed composite materials. Nearly all fiber materials are denser than the resins, with the exception of wood fibers, UHMWPE fibers, and certain other synthetic polymer fibers which have density less than water. A comparison of the density of select fibers is shown in Table 15.

#### 3.2.2. Strength

##### Tensile Strength

**Figure 7:**
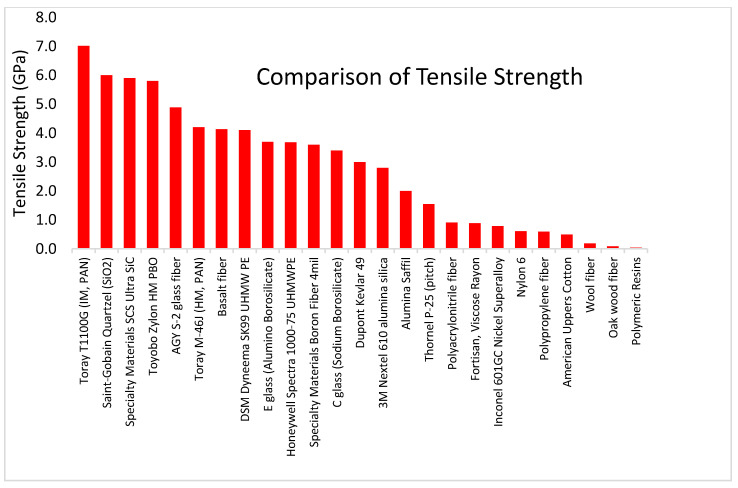
Comparison of Tensile Strength of Select Fibers and Resins.

Fiber tensile strength is an important characteristic to consider, and perhaps the most important aspect of reinforcing additive manufacturing materials that are pulled in tension. By themselves, the reinforcing fibers have a tensile yield strength one or two orders of magnitude stronger than the yield strength of associated composite resin matrix materials. Figure 7 and Table 16 compare the yield strength of reinforcing fibers and resin materials. Intermediate modulus carbon fibers from PAN have the highest tensile strength of all fibers, ranging from 6.2 to 7.0 GPa.

However, the higher tensile strength of short-cut fibers in FRAM materials does not linearly correlate to higher tensile strength of printed materials the same way fiber density changes printed material density. The resulting tensile strength of printed composite material is significantly reliant on the surface interfacial bonding and slippage of fibers inside the resin. Imperfect bonding of fiber-resin surfaces significantly reduces the tensile strength of the composite material. Materials with limited bonding between fibers and resin may show tensile strength lower than material printed without fibers. This is due to incorporation of defects and stress concentration points in the printed material.

##### Compressive Strength

The compressive strength of fibers used in additive manufacturing is a topic that warrants further exploration. Composite materials under a flexural load have fibers in tension and compression. Ceramic, oxide, and mineral fiber categories are the strongest in compression. For polymeric fibers, the basic molecular and nanometer scale structure determines its ability to withstand compressive stress. The bidirectional network of hydrogen bonding in M5 PIPD liquid crystalline fibers results in exceptional compressive strength, as the M5 is noted as having the highest compressive strength of all polymeric fibers at 1.7 GPa [43]. Well-oriented polymer fibers can achieve compressive strengths nearly 30% of their inter-chain shear modulus [44]. M5 fibers are excellent candidates for use in ballistic protective fabrics due to their strength in compression. A comparison of fiber compressive strength is given in Table 17.

##### Interfacial Strength

The interfacial bonding between fibers and the matrix is a critical factor in determining the resulting strength of the printed material. If the fibers bond well with high interfacial shear strength, the strength of the printed material benefits from the strength of the fibers. However, if the fibers delaminate and separate from the resin at a lower shear stress, the fibers slide inside the resin and fail to transfer stress, resulting in resin failure. The interfacial shear strength is a complex arrangement of mechanical and chemical factors along the three dimensional interphase of a fiber and matrix. Five key factors determine the strength of a fiber’s bond with resin, including adsorption and wetting, interdiffusion and chemical reaction, electrostatic attraction, mechanical keying, and residual stresses [94].

Adsorption and wetting describes the adhesion created between two bodies, typically a solid and liquid body, by van der Waals forces represented in the summation of surface energies known as the Dupre’ equation. If the surface energy of the fibers is significantly higher than the surface energy of the molten flowing resin, a higher degree of adhesion will occur [94]. For example, glass fibers and carbon fibers with surface energies of 560 and 70 mJ/m^2^ respectively adhere well with polyester and epoxy resins, with 35 and 43 mJ/m^2^ respectively, while polyethylene fibers with a surface energy of only 31 mJ/m^2^ does not bond well with polyester or epoxy resins [94].

Chain entanglements of polymer chains between fiber and resin at the molecular level serves to increase the interdiffusion and adhesion between the fiber and resin. Chemical reactions can also take place between the fiber and resin to improve the adhesion, either purposely or naturally, through construction of new covalent, ionic, or metallic bonds between the fiber and resin [94]. Chemical bonds are the strongest determinant of interfacial bonding.

Electrostatic attraction contributes to interfacial shear strength. If the fiber and resin have slightly opposite electrical charges, the electrostatic force will serve to adhere the fiber and resin [94]. However, electrostatic forces are much weaker than chemical bonding, chain entanglements, and mechanical keying.

The fiber surface roughness is an important factor when considering the interfacial strength gained by the resin hardening around the fiber. Mechanical keying is most significant when considering shear stress and fiber sliding. Fibers with rough surfaces that are well wetted with resin will significantly resist debonding and sliding [94].

Thermoplastic resins typically have higher coefficients of thermal expansion than reinforcing fibers, resulting in residual tension in the matrix and residual compression in the fibers. The residual axial tension of resin encircling fibers acts as hoop stress which increases the interfacial bonding between the fiber and resin [94].

Interfacial bonding strength is a property held by a fiber-resin combination. Each fiber type may have a different interfacial shear strength in PETG, ABS, PLA, and other resins. There are several methods to test interfacial shear strength directly or indirectly. Transverse flexural testing of a unidirectional composite is a method to indirectly measure the fiber-matrix interfacial shear strength [95]. The interfacial shear strength of individual fibers can be measured by several techniques, including embedded single fiber tension, embedded single fiber compression, microdebond, single fiber push-out, single fiber pull-out, and the microbond (bead pull-off) test [96].

#### 3.2.3. Stiffness

**Figure 8:**
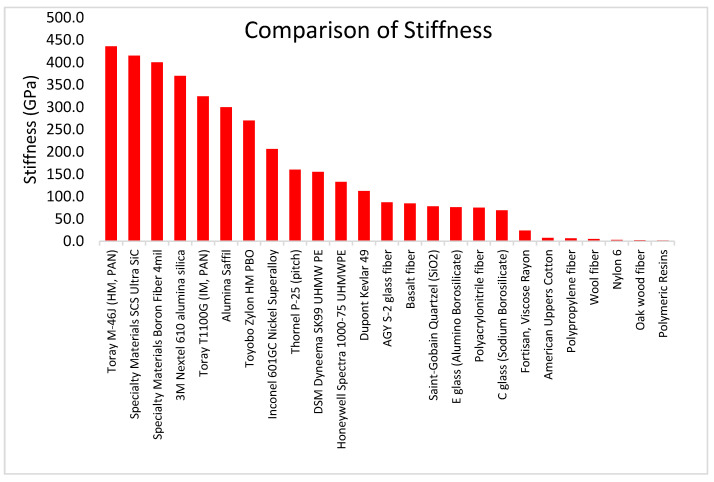
Stiffness of Selected Fibers and Resins (as reported in technical data sheets).

The ability of the fibers within composite materials to resist strain is an important characteristic. The graph below shows a comparison of the ability of fibers and resin to resist uniaxial stretching based on tensile modulus. Pitch-based carbon fibers show the highest values of specific stiffness, with 436 GPa for the Toray M-46 high modulus carbon fiber and 324 GPa for the Toray T1100G intermediate modulus carbon fiber. After carbon fibers, the next stiffest fiber materials are ceramic, mineral, PBO, metallic, and UHMWPE. Among the most elastic fibers are natural wood, synthetic polymers, and regenerated cellulose. Figure 8 compares the stiffness of selected fibers and resin materials.

**Figure 9:**
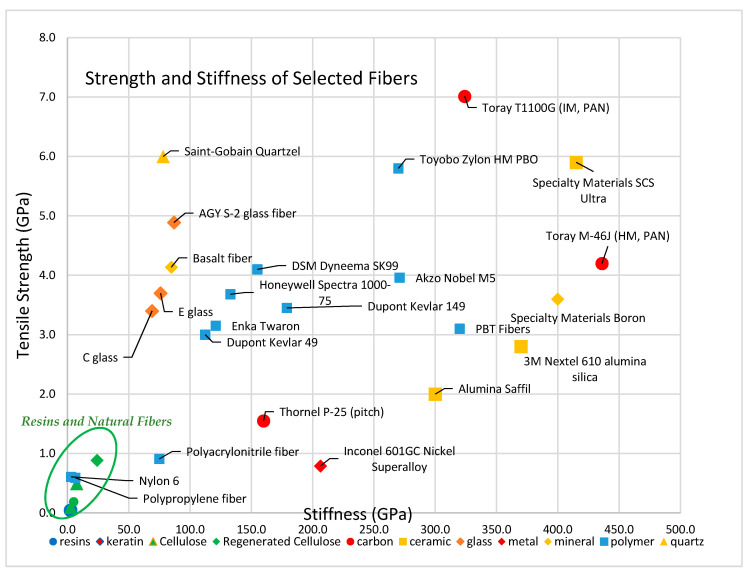
Comparison of Tensile Strength and Stiffness of Fibers and Resins.

Figure 9 graphs the tensile strength of selected fibers against the fiber stiffness in comparison to the strength and stiffness of resin matrix materials. The resins and natural fibers are graphed in Figure 2. Carbon, ceramic, metallic, mineral, glass, and polymer fibers are much stronger and stiffer than resins and natural fibers. Short-cut PAN-based carbon fibers are popular FRAM materials in the form of feedstock pellets, mixing with PLA, ABS, PETG, and other matrix materials [97].

#### 3.2.4. Flexibility and Flexural Rigidity

Fiber flexibility and fiber flexural rigidity are mechanical properties that represent the respective ease or resistance to fiber bending motion. Fiber flexibility can be expressed as k/M, where *M* is the moment required to bend a fiber with circular cross section to a given curvature and *k* is the reciprocal of the radius of curvature, as shown in Equation (1) [94]. The flexibility of a fiber is inversely related to its tensile modulus, shape factor, and fourth power of its diameter. Small variations in diameter relate to large changes in flexibility.

(1) $$\frac{k}{M}=\frac{64}{\pi Ed^{4}}$$

Fiber flexural rigidity can be considered the reciprocal of fiber flexibility. In classical beam theory, flexural rigidity of a beam is given as *EI*, where *E* is the elastic modulus in units of pascal, and I is the second moment of area in units of m^4^. The textile industry uses this same measurement for the flexural rigidity of fibers with specific tensile modulus and linear density [98,99]. If *η* represents the shape factor of the fiber, *ρ* the volumetric density, *c* the linear density, and *E_s_* the specific modulus (*E*/*ρ*), the flexural rigidity of a fiber is the product *EI* shown in Equation (2) in units of N·m^2^.

(2) $$EI=\frac{\eta E_{s}c^{2}}{4\pi \rho }$$

**Figure 10:**
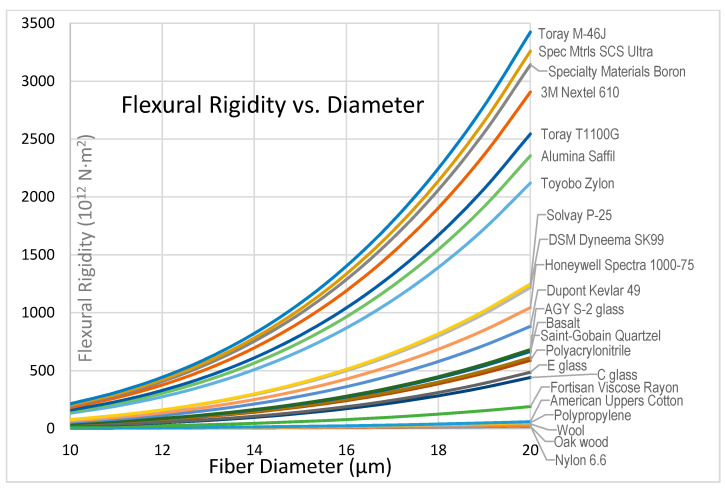
Comparison of Flexural Rigidity over a range of diameter sizes.

Using this calculation, the flexural rigidity of select fibers is shown in Figure 10, with fiber diameters ranging from 10 to 20 µm. Fibers with higher values of flexural rigidity have higher resistance to bending, while fibers with lower values have greater bending flexibility.

**Figure 11:**
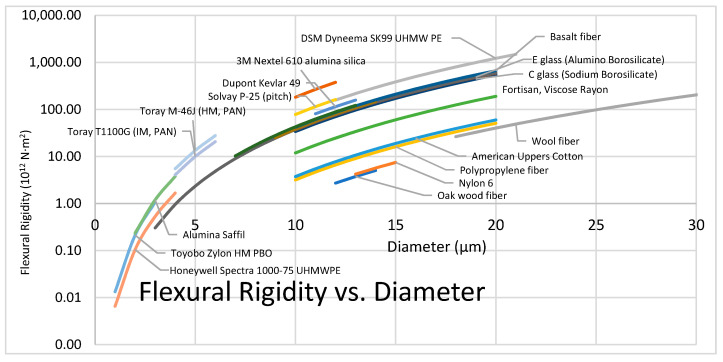
Flexural Rigidity at Actual Diameter Sizes.

Taking the most common diameter range for selected fibers, flexural rigidity is indicated in Figure 11 with logarithmic scale.

#### 3.2.5. Moisture Regain

Many additive manufacturing resins are hygroscopic, meaning they readily absorb moisture from the air. Nonhygroscopic filaments do not absorb moisture. The moisture absorption value is the maximum moisture content the material can absorb, expressed as a percentage of the water weight divided by the total weight. Table 18 shows a comparison of moisture absorption rates of resin filaments.

**Figure 12:**
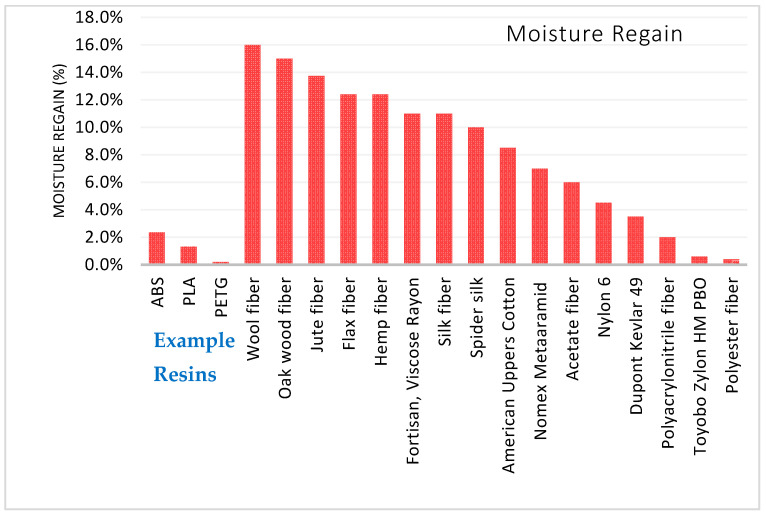
Moisture Regain of Selected Fibers and Resins.

The ability of fibers to absorb water is expressed as moisture regain, which is the percentage of the water weight divided by the dry fiber weight. Organic fibers tend to absorb moisture, while inorganic fibers are nonhygroscopic. Of the organic fibers, natural fibers absorb the most moisture, and by far the most absorbing fiber is wool. Wool fibers can absorb 16% to 17% of dry weight as water. Silk is another animal fiber that absorbs 10% to 11% moisture. Natural cellulose fibers absorb a significant amount of water as well. Wood fibers absorb up to 15% moisture, followed closely by Jute, Flax, Hemp, and Cotton fibers, which absorbs around 8.5% moisture. Regenerated cellulose fibers (acetate and rayon) absorb moisture, as well as certain synthetic polymer fibers, namely aramid fibers (Nomex and Kevlar) and polyamide fibers (nylon). The moisture regain of selected fibers is compared to resin materials in Figure 12.

### 3.3. Thermal Considerations

Designers must consider thermal characteristics of reinforcing fibers along with thermal properties of the matrix resin materials. The melting temperature, decomposing temperature, glass temperature, Coefficient of Thermal Expansion (CTE), and thermal conductivity are important characteristics to consider.

#### 3.3.1. Maximum Temperature

The nozzle temperatures required to melt the resin materials in the additive manufacturing process may have negative effects on reinforcing fibers. As shown earlier in Table 1, typical nozzle temperatures for common polymeric resins with short-cut fibers are 215 to 400 °C. Reinforcing short-cut fibers mixed in with the polymer resin matrix may melt or decompose during production at these temperatures. Nonpolymeric fibers such as ceramic, glass, quartz, mineral, basalt, metallic, and carbon have decomposing temperatures well above and are not affected by nozzle temperatures. Natural cellulose, regenerated cellulose, and keratin fibers, for example wool, cotton, wood, vegetal fibers, decompose if exposed to these temperatures in open air but are not significantly affected by short duration nozzle temperatures while mixed in resin. The degradation temperature of wool, silk, cotton, and rayon is 132 °C, 170 °C, 240 °C, and 205 to 240 °C respectively [100].

However, thermal shrinkage due to relaxation of stretched polymer chains can be a significant problem for synthetic polymer fibers at typical nozzle print temperatures. Macromolecules of polymer fibers are bound together by weak cohesive bonds and mechanical entanglements, as opposed to strong chemical bonds [100]. Thermal relaxation occurs at higher temperatures when cohesive bonds break and fiber backbone macromolecules gain mobility and slide past one another. Amorphous polymer fibers are characterized by glass transition temperatures at which molecular chains begin to detach and move around, as the fiber transitions from a hardened brittle glass-like structure to a soft rubbery structure. Below the glass transition temperature, macromolecule motion within the fiber is restricted to polymer side chains or slow conformational movements [101]. At temperatures above the glass transition temperature, amorphous polymer fibers undergo segmental mobility of backbone macromolecule chains, resulting in coiling, packing, and slipping [101]. Thermal relaxation in amorphous polymer fibers causes a degradation of tensile strength. Thermally stimulated shrinkage includes macroscopic contraction and decreased molecular orientation within the fiber [101]. Nylon, UHDPE, polypropylene, and PAN fibers have very low glass transition temperatures making these fibers vulnerable to additive manufacturing nozzle temperatures.

Crystalline polymer fibers are not affected by a glass transition point like amorphous polymer fibers and are defined instead by a melting point temperature at which their orderly crystalline molecular structure becomes disorderly and macromolecules move around in an amorphous viscous state. Kevlar para aramid fibers and Zylon PBO fibers are liquid crystalline polymeric fibers with a decomposition temperatures of 427 °C and 650 °C respectively which is high enough to avoid damage by typical nozzle printing temperatures. Fibers vulnerable to the range of additive manufacturing nozzle temperatures are shown in Table 19.

#### 3.3.2. Thermal Expansion

**Figure 13:**
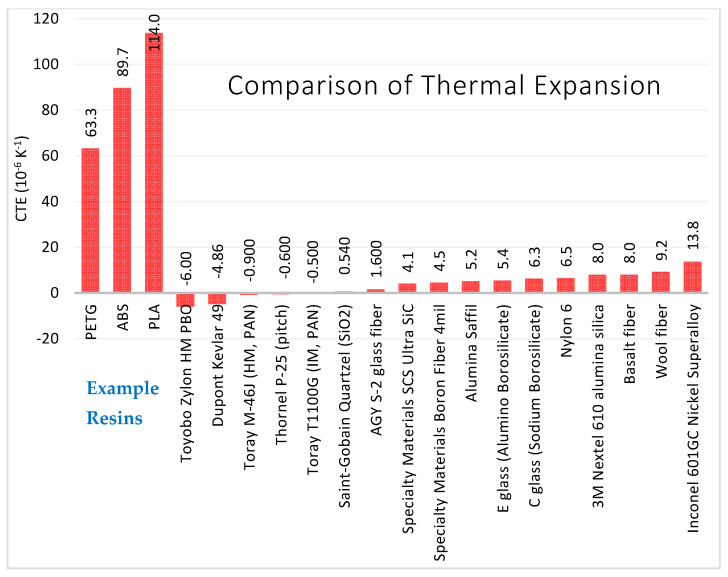
Comparison of Thermal Expansion.

The CTE indicates whether a material will expand or contract with a change in temperature. Matrix resins have very high CTEs as thermal expansion is dominated by intermolecular forces of polymer macromolecules. Compared to matrix resins, fibers have very low CTEs, while some fibers have negative CTEs indicating they contract when heated and expand when cooled. Fibers with low or negative CTE values may temper the thermal expansion and contraction of a resin matrix material, inducing static stress within the printed composite material as the composite is heated or cooled. A comparison of thermal expansion coefficients for selected fibers and resins is shown in Figure 13.

#### 3.3.3. Thermal Conductivity

**Figure 14:**
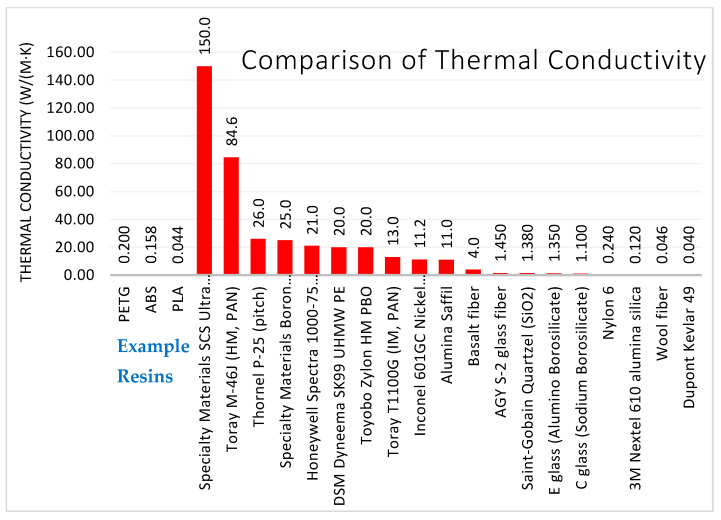
Comparison of Thermal Conductivity.

The ability of fibers to enhance or inhibit the conductive transfer of heat is a consideration for selecting fibers for additive manufacturing. Polymeric resin matrix materials typically have very low thermal conductivity. The selection of reinforcing fibers with high thermal conductivity may increase the thermal conductivity of the resulting composite. Ceramic silicon carbide fibers have the highest thermal conductivity of all fibers reviewed. The SCS Ultra lists a thermal conductivity of 150 W/(m·K), two orders of magnitude greater than potential matrix materials. Ceramic fibers, carbon fibers, boron fibers, and UHMWPE fibers all show high thermal conductivity. Wool, Kevlar, nylon, and glass fibers would have little effect on the resulting thermal conductivity of printed materials. Figure 14 compares the thermal conductivity for selected fibers in comparison to matrix resin materials.

## 4. Fiber Comparison Summary

Overall, the fibers that appear best suited for additive manufacturing include all types of carbon fibers, glass and quartz fibers, ceramic fibers, natural wood and cellulose vegetal fibers, basalt fibers, metallic fibers, and liquid crystalline polymeric fibers such as Kevlar. Designers wishing to maximize tensile strength while retaining flexibility should consider glass, quartz, and basalt fibers. Quartz fibers in particular shows high tensile strength with relatively low stiffness [102]. Glass fibers and basalt fibers show high strength and moderate stiffness. PAN based carbon fibers and nonoxide ceramic fibers show the highest combined tensile strength and stiffness. All fiber families have higher tensile strength and stiffness than the matrix resin materials. Designers wishing to minimize the weight of printed composite materials should consider natural cellulose fibers especially wood fibers. Designers wishing to add mass to a composite material should consider more dense fiber options especially metallic, mineral, and ceramic fibers.

If the printed material is intended for high moisture environments or intended to interact with water, designers might avoid hydrophilic fibers with high moisture regain values such as natural cellulose and keratin. If the intent is to design composite materials that resist expansion and contraction in changing temperature conditions, a designer might consider fibers with very low or negative CTE values. All fibers surveyed have CTE values less than the resin materials and will temper the resulting composite material expansion and contraction. Quartz, PBO, Kevlar, and carbon fibers in particular have very low or negative CTE values.

Below is a summary of comparison of mechanical and thermal characteristics for specific fibers using specific fibers to represent their categories. For example, the selection of American Uppers cotton is meant to represent all cotton fibers, while Toray M46J is meant to represent all similar high modulus PAN-based carbon fibers.

Natural cellulose, keratin, and regenerated cellulose fibers are suitable for use as reinforcing fibers in polymeric additive manufacturing processes. American Uppers cotton [30] is an organic, natural, cellulose, vegetal, cotton seed fiber. Cotton fibers are durable, elastic, and flexible. Cotton is hydrophilic and retains moisture with high regain. Cotton decomposes at 246 °C in air; however, short durations of such conditions do not damage the integrity of cotton fibers containing polymeric resins. Oak wood fibers [103] are organic, natural, cellulose, hard wood fibers that are lightweight, durable, and flexible. Oak wood fibers are the lightest of all fibers reviewed. Similar to cotton, oak wood fibers are hydrophilic with high moisture regain. Oak wood fibers decompose in air at 250 °C; however, this is likely not a problem for the short duration of polymeric additive manufacturing. Wood fibers are currently popular reinforcing material in additive manufacturing as described in the conclusion below. Jute, ramie, hemp, and flax fibers [29] are natural cellulose vegetal fibers well suited for additive manufacturing similar to wood fibers. The advantage of natural cellulose vegetal fibers as reinforcing compounds in additive manufacturing is their relative abundance and sustainability. Fortisan (rayon) [29] is a regenerated cellulose fiber that is elastic and flexible. Wool [31] is an organic, natural, keratin based animal fiber that is durable, and flexible. Wool decomposes in air at 222 °C although the short duration of printing wool fibers mixed in polymeric resin likely will not damage the fiber structure. Overall, natural fibers appear well suited for use as reinforcing compounds in FDM additive manufacturing processes.

Synthetic polymer fibers, however, present significant challenges as reinforcing compounds for additive manufacturing because of their susceptibility to high temperatures. DSM Dyneema SK99 [53] and Honeywell Spectra 1000-75 [57] are UHMWPE fibers that are lightweight, strong, stiff, and moderately rigid. However, Dyneema fibers melt at 144 °C and Spectra fibers at 147 °C, well below normal FDM printing nozzle temperatures. Dyneema and Spectra begin decomposing at 65 °C and have a “maximum use temperature” of 70 °C. The temperatures required to melt and flow polymeric matrix resins would damage the integrity of UHMWPE fibers. Nylon 6.6 and Perlon (nylon 6) [104] are organic synthetic polyamide fibers that are flexible and elastic. However, nylon 6.6 and nylon 6 fibers have glass transition temperatures at 70 °C and 47 °C and melt at 236 °C and 214 °C respectively. Polypropylene [29] is an elastic and flexible polyethylene fiber with a glass transition temperature of 0 °C, a decomposition temperature of 100 °C, and melting temperature of 162 °C. Polyacrylonitrile [105] is an acrylic polymer with fair strength and stiffness, and moderate rigidity. However, PAN fibers have a glass transition temperature of 95 °C and decompose at 300 °C. Polyamide, polyethylene, UHMWPE, and polyacrylonitrile fibers are not candidates for reinforcement of additive manufacturing materials because of their glass transition temperature and melting temperatures within the FDM nozzle range, which makes them susceptible to damage.

Liquid crystalline fibers are candidates for additive manufacturing. DuPont Kevlar 49 [47] is para aramid liquid crystalline fiber with moderate strength, stiffness, and bending flexibility. The negative CTE indicates Kevlar fibers contract when heated and expand when cooled which may induce stress and stability within a printed composite material. Kevlar fibers have the lowest thermal conductivity of all fibers reviewed. The crystalline nature of para aramid fibers results in higher glass transition and decomposing temperatures which enables successful reinforcing of additive manufacturing. Kevlar fibers decompose in air at 427 °C. Kevlar fibers are currently in use as long continuous reinforcing for additive manufacturing. Toyobo Zylon [46] is a high modulus polybenzoxazole synthetic polymer fiber. Zylon has high strength and stiffness. Similar to Kevlar fibers, the negative CTE of Zylon fibers indicates it contracts when heated and expands when cooling. Zylon fibers are candidates for additive manufacturing.

Carbon fibers are already in widely used as reinforcing compounds in additive manufacturing. Toray M-46J [64] is a high-modulus PAN-based carbon fiber with high stiffness and the highest flexural rigidity of all fibers reviewed. Toray M-46 has a low CTE and high thermal conductivity that may serve to temper composite material thermal expansion and contraction. Toray M-46 fibers are currently popular as short-cut reinforcing fiber in additive manufacturing applications today. Toray T1100G [64] is an intermediate-modulus PAN-based carbon fiber with very high strength, stiffness, and flexural rigidity. Similar to Toray M-46J, Toray T1100G has a low CTE and high thermal conductivity that may temper composite thermal expansion and contraction. PAN based carbon fibers are well suited for additive manufacturing applications. Solvay Thornel P-25 [106] is a pitch-based carbon fiber with high stiffness and moderate flexural rigidity. Similar to the PAN-based carbon fibers, pitch-based carbon fibers have low CTE and moderate thermal conductivity. The Solvay Thornel P-25 carbon fiber is currently a popular additive manufacturing reinforcing fiber.

Glass fibers are already widely used as reinforcing compounds for additive manufacturing. Saint-Gobain Quartzel [82] and JP Stevens Astroquartz II [83] are quartz fibers with exceptionally high tensile strength and moderate stiffness and flexural rigidity. Astroquartz and Quartzel have low CTE values which may temper composite material thermal expansion and contraction under heating and cooling. The AGY S-2 [107] in particular has high strength, high stiffness, high flexural rigidity, low thermal expansion, and low thermal conductivity. There are many E-glass [79] fibers currently in production as reinforcing compounds for additive manufacturing, including ECR-glass fibers represented by Binani SE1500 [85] and AGY Advantex [6]. C-glass [79], R-glass [6], A-glass [6], and D-glass [6] are all candidates for reinforcing fibers for additive manufacturing.

Ceramic fibers are well suited as reinforcing compounds for additive manufacturing, including oxide ceramics and nonoxide ceramics. Saffil [108] is an aluminum oxide ceramic staple fiber with moderate strength, stiffness, and flexural rigidity. Saffil has a low CTE and high thermal conductivity. 3M Nextel 610 [77] is another aluminum oxide ceramic fiber with very high stiffness and moderate tensile strength. The 3M Nextel 610 fiber has a very low thermal conductivity and very low CTE. The Specialty Materials SCS Ultra [72] is a nonoxide ceramic fiber made from silicon carbide with very high tensile strength, stiffness, and flexural rigidity. The SCS ultra has the highest thermal conductivity of all fibers reviewed, and a low CTE. Similar to Saffil, the high thermal conductivity and low CTE may transfer heat within the composite material without excessive expansion and contraction. The SCS Ultra, silicon carbide, and all ceramic fibers in general are great candidates for additive manufacturing.

Mineral and element fibers are also suitable for additive manufacturing. Specialty Materials Boron 4mil [109] is a mineral fiber that is lightweight, very stiff, and brittle. Boron fibers have low CTE and low thermal conductivity; however, they are are not easily produced through CVD and are expensive compared to other fibers. The CVD production of boron fibers results in relatively large diameter sizes, around 142 µm. Basalt [110] is a volcanic rock elemental fiber with high strength, stiffness, and moderate flexural rigidity. Basalt fibers have low CTE and moderate thermal conductivity. Basalt fibers are easily produced and plentiful, and are great candidates for additive manufacturing applications. Basalt fibers have already seen use in composite materials. Asbestos fibers are not candidates for additive manufacturing because of the health risks involved with airborne asbestos particles as described in Section 2.

Metallic fibers are well suited for reinforcing within additive manufacturing. Inconel 601GC Nickel Superalloy [111] fiber has the highest density of all fibers reviewed and would add mass to any composite printed material. The Inconel 601GC has moderate strength, stiffness, and flexural rigidity, with a high thermal conductivity and moderate CTE.

## 5. Conclusions

The production of fiber with reinforced additives will continue to grow in many industries, including aerospace, automotive, marine structures and vessels, civil engineering, transportation infrastructure, and buildings. An example of fiber reinforced additive manufacturing for marine vessels is the world’s largest 3D printed boat, the “3Dirigo”, produced by the University of Maine in 2019 [112]. The 3Dirigo measured 762 cm in length, weighed 2268 kg, and was printed with natural cellulose wood fibers from Maine’s vast forest resources [112]. As noted in this review, wood fibers have the lowest density of all fibers, which is important for buoyancy. Another benefit for bio-based additive manufacturing with wood fiber reinforcement is environmental sustainability.

This review conducted an overarching survey of the various categories of fibers to determine which may be appropriate for consideration as reinforcing materials in additive manufacturing. The complex interfacial characteristics between fibers and matrices are important for performance evaluations of the resulting composite materials. Extensive matrix-fiber interface studies, experimental testing, and computational modeling will greatly contribute to our understanding of how short-cut fibers contribute to the overall strength, flexibility, and stiffness of printed materials. Fiber selection for additive manufacturing remains a wide open field for research and development.

## Figures and Tables

**Table 1:** Examples of Commercially Available Matrix Resins with Short Cut Fibers.

Polymeric Resins		Fiber	Glass TransitionTemp (°C)	Extrusion Temp (°C)
PLA	Poly(L-lactic acid), polyactide [3]	Carbon, Glass	60	215
ABS	Acrylonitrile butadiene styrene [3]	Carbon, Glass	105	230
PETG	Poly(ethylene terephthalate) [3]	Carbon	80	245
PP	Polypropylene [3]	Carbon, Glass	−20	265
PA6	Nylon 6 [3]	Carbon, Glass	70	275
PA12	Nylon 12 [3]	Carbon	158	285
PC	Polycarbonate [3]	Carbon	143	300
PPS	Polyphenylene Sulfide [6]	Glass	90	309
PEI	Polyetherimide [3]	Carbon, Glass	217	385
PEKK	Polyketone [3]	Carbon	165	390
PEEK	Polyether ether ketone [3]	Carbon, Glass	143	400

**Table 2:** Vegetable Plant Fiber Types and Examples.

Seed	Stem (Bast)	Leaf	Husk/Fruit
Cotton, Kapok	Flax, Hemp, Jute, Ramie	Sisal, Abaca	Coconut (Coir)
Akon, Milk Wheat	Kenaf, Kudzu, Linden	Palm, Manila	Banana
Rice Husk	Milkweed, Nettle, Okra	Caraua	Agave

**Table 3:** Keratin Fiber Types.

Wool	Hair	Silk
Sheep, Alpaca, Angora Rabbit	Horse,	Spider silk
American Bison, Cashmere Goat	Camel	Silkworm silk
Mohair (Angora Sheep), Muskox		

**Table 4:** Density and Tensile Strength of Natural Fibers.

Natural Fiber	Protein Type	Density (g/cm^3^)	Tensile Strength (GPa)	Specific Strength (MY)
Spider silk [26,28]	Keratin	1.31	1.400	1.069
Ramie fiber [29]	Cellulose	1.50	0.885	0.590
Flax fiber [29]	Cellulose	1.54	0.831	0.540
Hemp fiber [29]	Cellulose	1.50	0.705	0.470
Cotton [30]	Cellulose	1.52	0.684	0.450
Silkworm silk [25]	Keratin	1.34	0.509	0.380
Jute fiber [29]	Cellulose	1.50	0.465	0.310
Wool fiber [31]	Keratin	1.31	0.185	0.141
Oak wood fiber [32]	Cellulose	0.74	0.090	0.122
Pine wood fiber [6]	Cellulose	0.35	0.078	0.223
Balsa fiber [6]	Cellulose	0.16	0.073	0.456

**Table 5:** Synthetic Polymer Fiber Types.

Amide	Polyester	Liquid Crystalline	Olefin	Common Polymers
Perlon	PET	Para-aramid	Polypropylene	PVA, PTFE
Nylon 6.6	Dacron	Meta-aramid	Polyethylene	Polyurethane
	Terylene	Aromatic Heterocycle, Copolyester	(HDPE, LDPE, UHMWPE),	Acrylic

**Table 6:** Density and Tensile Strength of Polyamide and Polyester Synthetic Polymer Fibers.

Amide Synthetic Fibers	Type	Density (g/cm^3^)	Tensile Strength (GPa)	Specific Strength (MY)
Perlon (nylon 6) [6]	Polyamide	1.14	0.578	0.507
Nylon (nylon 6.6) [6]	Polyamide	1.14	0.508	0.446
PET [35]	Polyester	1.38	1.133	0.821

**Table 7:** Density and Tensile Strength of Liquid Crystalline Synthetic Polymer Fibers.

Liquid Crystalline Synthetic Fibers	Type	Density (g/cm^3^)	Tensile Strength (GPa)	Specific Strength (MY)
Toyobo Zylon HM (PBO) [46]	Aromatic Heterocycle	1.56	5.800	3.718
Akzo Nobel M5 (PIPD) [44]	Aromatic Heterocycle	1.70	3.960	2.329
Dupont Kevlar 29 [47]	Para Aramid	1.44	3.617	2.512
Teijin Twaron HT [48]	Para Aramid	1.45	3.600	2.500
Teijin Technora [48]	Para Aramid	1.39	3.500	2.518
Dupont Kevlar 149 [47]	Para Aramid	1.47	3.450	2.347
Teijin Twaron HM [48]	Para Aramid	1.44	3.312	2.300
Teijin Twaron SM [48]	Para Aramid	1.44	3.240	2.250
Vectran HT (HBA/HNA) [49]	Copolyester	1.41	3.200	2.270
Vectran UM (HBA/HNA) [49]	Copolyester	1.40	3.000	2.143
Dupont Kevlar 49 [47]	Para Aramid	1.44	3.000	2.083
Vectran NT (HBA/HNA) [49]	Copolyester	1.40	1.100	0.786
Teijinconex [48]	Meta Aramid	1.38	0.860	0.623
Dupont Nomex 430 [50]	Meta Aramid	1.38	0.609	0.441
Polybenzimidazole fiber (PBI) [51]	Aromatic Heterocycle	1.43	0.341	0.238

**Table 8:** Density and Tensile Strength of Olefin Synthetic Polymer Fibers.

Olefin Synthetic Fibers	Type	Density (g/cm^3^)	Tensile Strength (GPa)	Specific Strength (MY)
DSM Dyneema SK99 UHMWPE [53]	Olefin	0.97	4.100	4.227
Honeywell Spectra 1000-75 UHMWPE [57]	Olefin	0.97	3.680	3.794
Honeywell Spectra 900 UHMWPE [57]	Olefin	0.97	2.585	2.670
Toyobo Tsunooga UHMWPE [58]	Olefin	0.97	1.400	1.433
Polypropylene fiber [29]	Olefin	0.91	0.591	0.649
Polyethylene fiber [13]	Olefin	0.90	0.477	0.530

**Table 9:** Density and Tensile Strength of Carbon Fibers.

Carbon Fiber	Type	Density (g/cm^3^)	Tensile Strength (GPa)	Specific Strength (MY)
Toray T1100G (IM) [64]	PAN-based	1.79	7.012	3.917
Hexcel IM10 [65]	PAN-based	1.83	6.826	3.813
Toray T1000G (IM) [64]	PAN-based	1.80	6.371	3.539
Teijin Tenax IMS65 [66]	PAN-based	1.78	6.000	3.371
Hexcel IM7 [65]	PAN-based	1.78	5.688	3.196
Toray T800H (IM) [64]	PAN-based	1.81	5.490	3.033
Mitsubishi TRH50 [67]	PAN-based	1.82	5.300	2.912
Mitsubishi HS40 [67]	PAN-based	1.85	4.610	2.492
Solvay Thornel T650/35 [68]	PAN-based	1.78	4.275	2.415
Hexcel AS4 [65]	PAN-based	1.79	4.447	2.483
Toray M-46J (HM) [64]	PAN-based	1.84	4.200	2.283
Teijin Tenax UMS40 [66]	PAN-based	1.79	4.700	2.626
Toray M60J (HM) [64]	PAN-based	1.93	3.820	1.979
Mitsubishi K13C2U (EHM) [67]	Pitch-based	2.20	3.800	1.727
Mitsubishi K13D2U [67]	Pitch-based	2.20	3.700	1.682
Nippon YSH-70A-60S [69]	Pitch-based	2.14	3.630	1.696
Mitsubishi K1352U [67]	Pitch-based	2.12	3.600	1.698
Toray T300 [64]	PAN-based	1.76	3.530	2.006
Thornel P-120 (UHM) [6]	Pitch-based	2.13	2.400	1.127
Solvay P-25 [68]	Pitch-based	1.95	1.380	0.707
Solvay P-55 [68]	Pitch-based	2.10	1.380	0.657
Nippon XN-05-30S [69]	Pitch-based	1.65	1.100	0.667

**Table 10:** Density and Tensile Strength of Ceramic Oxide Fibers.

Nonoxide Ceramic Fiber	Type	Density (g/cm^3^)	Tensile Strength (GPa)	Specific Strength (MY)
Specialty Materials SCS Ultra [72]	Silicon Carbide	2.89	5.900	1.916
Avco 5.6mil [73]	Silicon Carbide	3.07	3.792	1.235
Avco 4.0mil [73]	Silicon Carbide	3.30	3.792	1.149
Specialty Materials SCS6 [72]	Silicon Carbide	3.08	3.450	1.150
UBE Industries Tyranno ZMI [74]	Silicon Carbide	2.48	3.400	1.371
UBE Industries Tyranno LoxM [74]	Silicon Carbide	2.48	3.300	1.331
UBE Industries Tyranno S [74]	Silicon Carbide	2.35	3.300	1.404
Nippon Carbon/Nicalon NL-200/201 [74]	Silicon Carbide	2.55	3.000	1.176
COI Ceramics/Sylramic-iBN	Silicon Carbide	3.00	3.000	1.000
Nippon DC HVR Nicalon [74]	Silicon Carbide	2.36	2.962	1.255
Nippon Carbon/Hi-Nicalon [74]	Silicon Carbide	2.74	2.800	1.022
UBE Industries Tyranno SA3 [74]	Silicon Carbide	3.10	2.800	0.903
Nicalon [6]	Silicon Carbide	2.55	2.760	1.082
Boron Carbide [73]	Boron Carbide	2.00	2.758	1.379
COI Ceramics/Sylramic [74]	Silicon Carbide	2.95	2.700	0.915
Nippon Carbon/Hi-Nicalon S	Silicon Carbide	3.10	2.600	0.839
Boron Nitride [73]	Boron Nitride	1.80	2.068	1.149

**Table 11:** Density and Tensile Strength of Ceramic Oxide Fibers.

Ceramic Oxide Fiber	Density (g/cm^3^)	Tensile Strength (GPa)	Specific Strength (MY)
3M Nextel 610 alumina silica [77]	3.90	2.800	0.718
Alumina Saffil [6]	3.30	2.000	0.606
Hiltex ALF G2 [78]	2.80	1.900	0.679
Hiltex ALF F2 [78]	2.90	1.800	0.621
Hiltex ALF E3 [78]	3.00	1.700	0.567
Hiltex ALF FB3 [78]	3.00	1.750	0.583
Nitivy ALF [74]	2.90	2.000	0.690
3M Nextel 550 [6]	3.03	2.000	0.660
3M Nextel 440 [6]	3.00	1.840	0.613
3M Nextel 720 [6]	3.40	1.940	0.571
Dupont Alumina SiO2 [73]	3.70	1.896	0.512
Sumitomo Altex Alumina [74]	3.30	1.800	0.545
Dupont Alumina FP-2 [74]	3.70	1.724	0.466
3M Nextel 312 Alumina [6]	2.80	1.630	0.582
Dupont Alumina FP-1 [74]	3.70	1.379	0.373

**Table 12:** Density and Tensile Strength of Glass Fibers.

Glass Fiber	Type	Density (g/cm^3^)	Tensile Strength (GPa)	Specific Strength (MY)
Saint-Gobain Quartzel [82]	Pure Silica (Quartz)	2.20	6.000	2.727
J.P. Stevens Astroquartz II [83]	Pure Silica (Quartz)	2.20	6.000	2.727
AGY S2-glass fiber [84]	Magnesium Borosilicate	2.46	4.890	1.988
S-glass fiber [6]	Alumino Silicate	2.49	4.585	1.841
R-glass fiber [6]	Alumino Silicate	2.54	4.135	1.628
AGY Advantex (ECR) [6]	Calcia Silicate	2.62	3.750	1.431
E-glass fiber [6]	Alumino Borosilicate	2.57	3.620	1.409
AGY S3-glass fiber [84]	Alumino Silicate	2.83	3.338	1.180
C-glass fiber [6]	Sodium Borosilicate	2.54	3.310	1.303
A-glass fiber [6]	High alkali	2.44	3.310	1.357
Binani SE1500 (ECR) [85]	Calcia Silicate	2.62	2.500	0.954
AR2-glass fiber [6]	Zirconia and Potash	2.74	2.500	0.912
D-glass fiber [6]	Boron Trioxide	2.11	2.415	1.145

**Table 13:** Asbestos Fiber Types.

Serpentine Asbestos	Amphiboles Asbestos
Chrysotile	Amosite (asbestos grunerite), Crocidolite, Tremolite, Anthophyllite, Actinolite

**Table 14:** Isotropic and Anisotropic Fibers.

Isotropic Fibers	Anisotropic Fibers
Glass, Quartz, Ceramic, Metallic	Cellulose, Keratin, Carbon, Mineral, Synthetic Polymer

**Table 15:** Density of Fiber Families and Polymeric Resins.

Fiber Family	Density (g/cm^3^)
Cellulose Fibers	0.14 to 1.54
Polymer Fibers	0.90 to 1.54
Polymeric Resins	1.07 to 1.29
Keratin Fibers	1.30 to 1.34
Regenerated Cellulose	1.25 to 1.52
Carbon Fibers	1.74 to 2.20
Glass and Quartz Fibers	2.20 to 2.60
Ceramic Fibers	1.80 to 3.90
Mineral Fibers	2.64 to 2.70
Metallic Fibers	1.74 to 8.92

**Table 16:** Tensile and Specific Strength of Fiber Families and Resin.

Fiber Family	Yield Strength (GPa)	Specific Strength (MY)
Carbon Fibers	2.20 to 7.00	1.20 to 4.00
Glass and Quartz Fibers	2.00 to 6.00	0.78 to 2.72
Polymer Fibers	0.33 to 5.80	0.27 to 3.80
Ceramic Fibers	1.40 to 5.90	0.40 to 1.90
Mineral Fibers	2.50 to 4.80	1.00 to 1.80
Metallic Fibers	0.22 to 2.21	0.03 to 0.28
Keratin Fibers	0.19 to 1.40	0.14 to 1.07
Regenerated Cellulose	0.17 to 0.89	0.13 to 0.59
Cellulose Fibers	0.07 to 0.89	0.12 to 0.59
Resin Material (PLA, PETG, ABS)	0.04 to 0.05	0.03 to 0.04

**Table 17:** Compressive Strength of Fibers.

Type	Fiber	Density (g/cm^3^)	Compressive Strength (GPa) [7]	Tensile Strength (GPa)
Alumina (Oxide Ceramic)	3M Nextel 610	3.90	6.90	2.80
Boron (Mineral, CVD)	Spec. Materials	2.6	5.90	3.60
Silicon Carbide (Nonoxide Ceramic)	Nicalon HI COI	2.55	3.10	2.80
PIPD (Aromatic Heterocycle)	Akzo Nobel M5	1.70	1.70	3.96
Carbon (PAN, High Modulus)	Toray M60J	1.93	1.67	3.82
S-Glass (Alumino Silicate)	AGY S2-Glass	2.46	1.10	4.89
Carbon (Pitch)	Solvay Thornel P100	2.13	0.48	2.40
Para Aramid	Dupont Kevlar 149	1.47	0.46	3.45
Polybenzazole (LC Synthetic)	Toyobo Zylon PBO	1.56	0.41	5.80
UHMWPE (Olefin)	Spectra 1000	0.97	0.17	3.68
Polyamide	Nylon 6	1.14	0.10	0.61
Polyester	PET	1.39	0.09	1.20

**Table 18:** Moisture Absorption of Resin Filaments.

Hygroscopic Filaments (Moisture Absorption)	Nonhygroscopic Filaments
PLA (1.3%), ABS (2.3%), PC (0.7%), PETG (0.2%), PET (0.8%)	PVC, Polypropylene, Polyethylene, Polystyrene

**Table 19:** Fibers with Low Glass Transition, Melting, or Decomposing Temperatures.

Fiber	Tg (°C)	Tm (°C)	Td (°C)
Honeywell Spectra (UHMWPE)	-	147	65
DSM Dyneema (UHMWPE)	-	144	65
Polypropylene (polyethylene)	0	162	-
Nylon 6 (polyamide)	47	214	-
Nylon 6.6 (polyamide)	70	236	-
Polyacrylonitrile	140	300	300
Kevlar (para aramid)	321	-	427
Zylon (PBO)	-	-	650
Cotton (Natural Cellulose)	-	-	246
Oak wood (Natural Cellulose)	-	-	250
Silk (Keratin)	-	-	170
Wool (Keratin)	-	-	132
Viscose Rayon (Regenerated)	-	-	240

**Figure A1:**
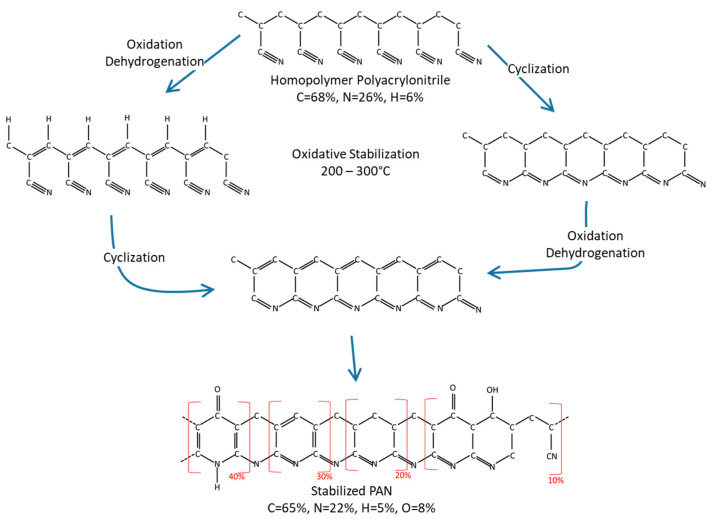
Stabilization of Polyacrylonitrile Fibers.

**Figure A2:**
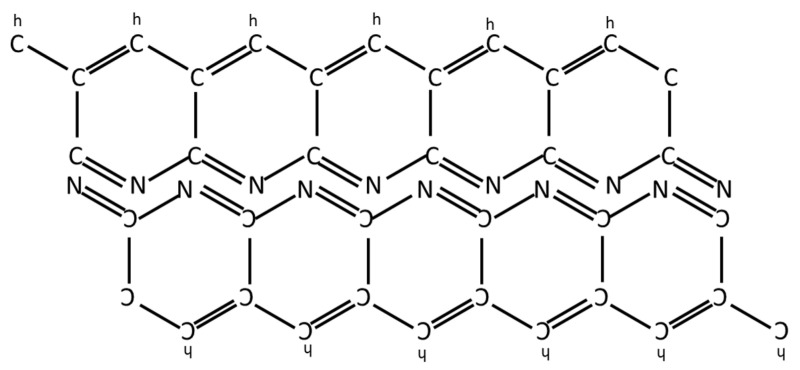
Nitrogen Reduction of Polyacrylonitrile Fibers.

**Figure A3:**
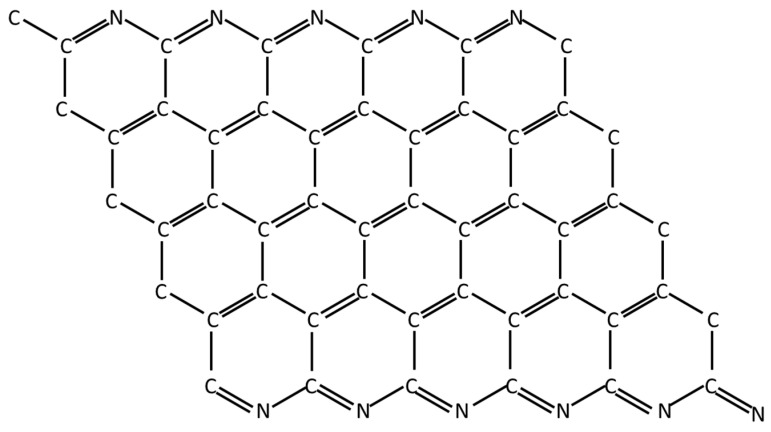
Carbonization of Polyacrylonitrile.

## Data Availability

The data presented in this study are available in referenced materials.

## Appendix A. The Family of Fibers

**Table A1:** Categorization of the Family of Fibers.

Family of Fibers	Organic	Natural	Cellulose	Plants (seed, stem, leaf, husk) (cotton, ramie, jute, hemp, flax, abaca)
				Trees (pulp of hardwood or softwood)
			Regenerated Cellulose	Viscose Rayon (Fortisan)
				Acetate, Triacetate
				Polylactic Acid
			Animal	Keratin (silk, wool, alpaca, angora, camel, cashmere, mohair)
		Synthetic	Amide	Nylon, Perlon
			Liquid Crystalline	Para-aramid (Kevlar, Twaron)
				Meta-aramid (Nomex)
				PBO, PBI
				HBA/HNA copolyester, Vectran
			Olefin	Ultra High MW Polyethylene (UHMWPE) (Spectra, Dyneema, IZANAS, Tsunooga)
				High Density Polyethylene (HDPE)
				Low Density Polyethylene (LDPE)
				Polypropylene (Typar, Tekton)
			Common	Acrylic, Polyacrylonitrile (PAN)
				Polyimide (P84)
				Polystyrene
				Polyvinylalcohol (PVA) (Nycon)
				Polyurethane (Spandex)
				Polytetrafluorethylene (PTFE) (Teflon)
	Inorganic	Element	Carbon	PAN based high modulus
				PAN based intermediate modulus
				Pitch based
				Graphite, activated carbon
			Volcanic Rock	Basalt
			Mineral	Boron
				Asbestos
			Metallic	Aluminum
				Steel
				Nickel and nickel alloys
				Titanium, Brass
		Ceramic	Nonoxide	Silicon Carbide, Boron Carbide
			Oxide	Alumina-silica
			Quartz	Quartzel, Astroquartz, pure silica
		Glass	S-Glass	S, S2, S3-glass
			E-Glass	E glass (Alumino Borosilicate), ECR
			C-Glass	C-glass (Sodium Borosilicate)
			B-Glass	B-glass
			A-Glass	A-glass, AR1, AR2

**Table A2:** Overall Fiber Comparison.

Fiber	Tensile Strength	Tensile Modulus	Bending	Moisture Regain	Nozzle Temp	Thermal Expansion	Thermal Conductivity
American Uppers Cotton [30] (Organic, Natural, Cellulose, Vegetable, Plant, Seed)	Fair	Elastic	Flexible	Very High	Safe	Low	Very Low
Oak wood fibers [103] (Organic, Natural, Cellulose, Hard Wood)	Fair	Elastic	Flexible	Very High	Safe	Low	Very Low
Fortisan (regenerated rayon) [29] (Organic, Natural, Regenerated Cellulose)	Fair	Elastic	Flexible	Very High	Safe	Low	Low
Wool [31] (Organic, Natural, Animal, Keratin)	Fair	Elastic	Flexible	Very High	Safe	Low	Very Low
DSM Dyneema SK99 [53] (Organic, Synthetic Polymer, UHMWPE)	High	Stiff	Moderate	None	Melt	Negative	High
Honeywell Spectra 1000-75 [57] (Organic, Synthetic Polymer, UHMWPE)	High	Stiff	Moderate	None	Melt	Negative	High
Nylon 6/6.6 [104] (Organic, Synthetic Polymer, Polyamide)	Fair	Elastic	Flexible	High	Melt	Low	Very Low
Dupont Kevlar 49 [47] (Organic, Syntetic Polymer, Polyamide Para aramid)	Moderate	Stiff	Moderate	Moderate	Near Max	Negative	Lowest
Toyobo Zylon HM [46] (Organic, Syntetic Polymer, Polybenzoxazole)	Very High	Stiff	Rigid	Moderate	Near Max	Negative	High
Polyacrylonitrile [105] (Organic, Synthetic Polymer, Acrylic)	Fair	Stiff	Moderate	Moderate	Melt	Low	Low
Polypropylene [29] (Organic, Synthetic Polymer, Polypropylene)	Fair	Elastic	Flexible	None	Melt	Low	Low
Toray M-46J (HM, PAN) [64] (Inorganic, Carbon, PAN-based)	High	Most Stiff	Most Rigid	None	Safe	Negative	Very High
Toray T1100G (IM, PAN) [64] (Inorganic, Carbon, PAN-based)	Very High	Stiff	Rigid	None	Safe	Very Low	High
Solvay Thornel P-25 [106] (Inorganic, Carbon, Pitch-based)	Fair	Stiff	Moderate	None	Safe	Very Low	High
Spec Mtrls Boron 4mil [109] (Inorganic, Mineral)	Moderate	Very Stiff	Highly Rigid	None	Safe	Low	High
Basalt [110] (Inorganic, Volcanic Rock)	High	Stiff	Moderate	None	Safe	Low	Moderate
Inconel 601GC Nickel Superalloy [111] (Inorganic, Metallic, Steel Alloy)	Fair	Stiff	Rigid	None	Safe	Moderate	High
Saffil [108] (Inorganic, Ceramic, Oxide)	Moderate	Stiff	Rigid	None	Safe	Low	High
3M Nextel 610 Al Si [77] (Inorganic, Ceramic, Nonoxide)	Moderate	Very Stiff	Rigid	None	Safe	Low	Very Low
Spec Mtrls SCS Ultra SiC [72] (Inorganic, Ceramic, Nonoxide)	Very High	Very Stiff	Highly Rigid	None	Safe	Low	Highest
Saint-Gobain Quartzel (SiO2) [82] (Inorganic, Ceramic, Quartz)	Very High	Stiff	Moderate	None	Safe	Very Low	Low
AGY S2-glass fiber [107] (Inorganic, Ceramic, Glass)	High	Stiff	Moderate	None	Safe	Very Low	Low
E-glass [79] (Inorganic, Ceramic, Glass)	High	Stiff	Moderate	None	Safe	Low	Low
C-glass [79] (Inorganic, Ceramic, Glass)	Moderate	Stiff	Moderate	None	Safe	Low	Low

## Appendix B. Explanation of Terminology and Units

(An)isotropic. Isotropic refers to a material property that is the same numerical value in all directions, while anisotropic is defined as having differing numerical values in differing directions.

Chopped Strands. Continuous filament fibers cut into various lengths from 3 to 50 mm, for the purpose of mixing into composite materials.

Family of Fibers. The phrase “family of fibers” is meant to encompass all types of fibers, including textile fibers and industrial fibers, which may have use for additive manufacturing. Most fibers considered discussed in this review are considered textile fibers; however, many, such as basalt, boron, wood, and oxide fibers, are not considered textile fibers. The “family of fibers” is meant to be inclusive of all fibers.

Fiber. An elongated material with a uniform diameter or cross sectional shape, generally less than 250 μm in width, with an aspect ratio generally above 100.

Filament. A filament is a long continuous fiber.

Flexibility. Fiber flexibility is used as a generic term to represent the inverse of rigidity in terms of bending moment. High rigidity corresponds to low flexibility and vice versa.

Glass Transition. When heated, the temperature at which an amorphous polymer transitions from glassy brittle state to a viscous rubbery state. Crystalline polymers are only defined by a melting temperature, while the amorphous parts of semi crystalline polymers have a glass transition temperature.

Modulus. Modulus is used as a generic term to represent the tensile modulus of a material, also known as Youngs modulus.

Moisture Regain. Moisture regain is used to represent the amount of water absorbed by a fiber in standard conditions compared to the fibers dry weight. The dry weight is determined by weighing the fiber equilibrated at 105 °C. The wet weight is determined by weighing the fiber equilibrated at 22 °C and 65% relative humidity. The water weight is the remaining weight after the dry weight has been subtracted from the wet weight. The moisture regain is the water weight divided by the dry weight. Similarly the water content is defined as the water weight divided by the wet weight.

Rigidity. Rigidity is used as a generic term to represent the fiber flexural rigidity as described by the textile industry. Rigidity is the resistance to bending defined as the bending moment required to bend the fiber to unit curvature. Rigidity represents flexural stiffness of the fiber.

Roving. Rovings are fine fibrous strands intended for spinning into yarns or threads.

Spinning. Spinning is the process used to produce fibers and filaments from glass, metals, polymers, and ceramics by extrusion. There are many types of spinning methods, including melt spinning, solution spinning, dry spinning, wet spinning, dry-wet spinning, centrifugal spinning, and electrospinning.

Staple Fiber. In the textile industry, a fiber that has been cut to a specific length from 10 to 400 mm, with a naturally wavy property conducive to spinning together into yarn. Staple fibers also are used extensively in the construction industry as insulation.

Stiffness. Fiber stiffness is used throughout as a generic term to represent tensile stiffness of a fiber pulled in tension, as represented by the fiber’s Young’s modulus or modulus of elasticity.

Strength. Fiber strength is used as a generic term to represent tensile and compressive strength. Tensile strength is a representation of average maximum effective yield strength of a fiber pulled in tension to failure. Some fibers yield before fracture while other fibers fracture suddenly. For comparison of fiber types, tensile strength is taken as the yield strength for fibers that yield prior to fracture. Compressive strength is taken as the yield strength of a fiber subjected to compression loading.

Whisker. A tiny fiber, generally a single crystal in width or diameter of 1 to 2 μm and length of 1 to 2 mm.

**Table A3:** Units of measurement used throughout.

Symbol	Name	Dimension	Derivation
m	meter	length	base unit
cm	centimeter	length	0.01 m
mm	milimeter	length	0.001 m
μm	micrometer	length	10^−6^ m
nm	nanometer	length	10^−9^ m
g/cm^3^	grams per cubic centimeter	density	0.001 kg⋅m^−3^
GPa	gigapascal	stress	10^9^ kg⋅m^−1^⋅s^−2^
MPa	megapascal	stress	10^6^ kg⋅m^−1^⋅s^−2^
MY	megaYuri	specific strength	10^6^ m^2^⋅s^−2^
m/min	meters per minute	linear rate	0.01667 m⋅s^−1^
W/(m⋅K)	watts per meter Kelvin	thermal conductivity	kg⋅m⋅K^−1^⋅s^−3^
CTE	coefficient of thermal expansion	strain per rate of temperature change	K^−1^

## Appendix C. Linear Density, Specific Strength, and Tenacity

Fiber strength and stiffness are key mechanical properties for the selection of fiber materials for additive manufacturing. Other important mechanical properties include weight, density, and thermal conductivity. Optimization parameters include filter media that is strong, resilient, lightweight, and able to withstand high temperature environments.

Linear Density is an important fiber measurement that compares the mass of a fiber to its unit length, while volumetric density compares the mass to its volume. The textile industry over the past several hundred years developed standards to evaluate and compare threads and yarns using “denier” (de) and “tex.” One denier has a linear density of one gram mass per 9000 m of fiber. With fiber and filament synonymous, the textile industry refers to denier per filament as “dpf”.

The tex is a measurement of fiber linear density as one gram mass per 1000 m of fiber, while a decitex (or dtex) represents one gram of mass per ten kilometers of fiber. Although not one of the 22 specially named derived units, the tex is recognized by the international system of units and is the most common SI representation of fiber linear density. One tex is equal to 10^−6^ kg/m of fiber.

Typical measurements of material strength are given in units of force per cross sectional area, such as megapascals (MPa). However, this convention of measurement works poorly with fibers since many fibers have hollow cores and irregular shapes which makes it difficult to measure cross sectional area and density. Specific strength is a better convention to measure fiber strength, defined as the breaking force of a fiber compared to its linear density, or the force required to make a fiber break compared to its denier or tex. The textile industry refers to the measurement of this characteristic the tenacity of the fiber. Five measurements of specific strength include megaYuri (MY), newtons per tex (N/tex), grams per denier (g/den), megapascals per density MPa/(g/cm^3^), and gigapascals per density GPa/(g/cm^3^).

The international space elevator consortium recognized the “Yuri” as a measure of specific strength for cable materials and recommended its recognition by the SI community; however, at this time, the Yuri is not recognized in the SI convention [113] A Yuri is the yield stress of a cable per unit density written in basic SI units, as one pascal of stress per unit density of one kilogram per cubic meter. Dimensionally, the Yuri has units of m^2^·s^−2^. Equation (A1) demonstrates the cancelation of cross sectional areas from the stress and density components, leaving just the breaking force divided by linear density.

(A1) $$1\;\mathrm{Yuri}=\frac{\mathrm{Pa}}{\frac{\mathrm{kg}}{m^{3}}}=\frac{\frac{N}{m^{2}}}{\left(\frac{\mathrm{kg}}{m}\right)\left(\frac{1}{m^{2}}\right)}=\frac{N}{\frac{\mathrm{kg}}{m}}=\frac{N\;m}{\mathrm{kg}}=\frac{\left(\frac{\mathrm{kg}\;m}{s^{2}}\right)\;m}{\mathrm{kg}}=\frac{m^{2}}{s^{2}\;}$$

The textile industry measures tenacity as grams per denier (gpd) in which breaking force is implicit. Grams of mass multiplied by the acceleration of gravity reveals breaking force as gram-force newtons. Equation (A2) shows the conversion between gpd and Yuri.

(A2) $$\mathrm{gpd}=\frac{g}{\mathrm{den}}=\frac{g\;\left(9.80665\frac{m}{s^{2}}\right)}{\frac{g}{9000\;m}}=88,260\frac{m^{2}}{s^{2}\;}=88,260\;\mathrm{Yuri}$$

The textile industry also uses newtons per tex to measure the specific strength of fibers, which is equivalent to 10^6^ m^2^·s^−2^, one million Yuris, a megaYuri, as shown in Equation (A3). The units work out such that one N/tex is equivalent to one megaYuri, which is equivalent to 11.33 gpd. The textile industry sometimes uses cN/dtex as in centiNewton per deciTex, or 0.01N/0.1Tex. One N/tex is equivalent to ten cN/dtex.

(A3) $$\frac{N}{\mathrm{tex}}=\frac{\frac{\mathrm{kg}\;m}{s^{2}}}{\frac{g}{1000\;m}}=\frac{\frac{1000\;g\;m}{s^{2}}}{\frac{g}{1000\;m}}=1,000,000\frac{m^{2}}{s^{2}\;}=\mathrm{megaYuri}=11.33\;\mathrm{gpd}$$

The construction industry measures specific strength of fibers as the breaking strength of the fiber per volumetric density, as megapascals or gigapascals divided by grams per cubic centimeter. The units work out such that one MPa/(g/cm^3^) is 1000 Yuris, and one GPa/(g/cm^3^) is the same as one N/tex as well as one megaYuri, as shown in (A4) and (A5).

(A4) $$\frac{\mathrm{MPa}}{\frac{g}{{\mathrm{cm}}^{3}}}=\frac{1,000,000\;\frac{N}{m^{2}}}{\left(\frac{\mathrm{kg}}{1000}\right)\left(\frac{1,000,000}{m^{3}}\right)}=1000\frac{m^{2}}{s^{2}\;}=1000\;\mathrm{Yuri}$$

(A5) $$\frac{\mathrm{GPa}}{\frac{g}{{\mathrm{cm}}^{3}}}=\frac{1,000,000,000\frac{N}{m^{2}}}{\left(\frac{\mathrm{kg}}{1000}\right)\left(\frac{1,000,000}{m^{3}}\right)}=1,000,000\frac{m^{2}}{s^{2}\;}=\frac{N}{\mathrm{tex}}=\mathrm{megaYuri}$$

Table A3 shows equivalent units of measurement for fiber tenacity or specific breaking strength. One MegaYuri is equivalent to one newton per tex and one gigapascal per g/cm^3^.

**Table A4:** Equivalent units of measurement for fiber tenacity.

Equivalent Value	Units of Specific Strength (SI Base Units of m^2^/s^2^)
1,000,000	Yuri
1	megaYuri (MY)
1	Newtons per tex (N/tex)
1	GPa/(g/cm^3^)
1000	MPa/(g/cm^3^)
11.33	grams per denier (g/den)

Fiber breaking length is another common measurement used by the textile industry, which can be thought of as the maximum length a fiber could hang from one end without breaking under its own weight. Equating the force of gravity as the product of mass and acceleration, the gravitational constant at the instant of fiber break represents the breaking force divided by the mass. The fiber breaking force, mass, and cross sectional area cancel when dividing the specific strength by the gravitational acceleration constant, which reveals the breaking length.

(A6) $$\frac{\mathrm{Specific}\;\mathrm{Strength}}{\mathrm{Gravitational}\;\mathrm{Constant}}=\frac{\frac{\mathrm{Pa}}{\mathrm{kg}/m^{3}}}{a}=\frac{\frac{\left(\frac{N}{m^{2}}\right)}{\left(\frac{\mathrm{kg}}{m^{2}\cdot m}\right)}}{\left(\frac{N}{\mathrm{kg}}\right)}=m=\mathrm{breaking}\;\mathrm{length}$$

## Appendix D. Composition of Fibers

**Table A5:** Composition of quartz, glass, and basalt fibers.

	Quartz	Glass								Basalt
		D	A	C	S2	AR	ECR	R	E	
Density (g/cm^3^)	2.15	2.1	2.44	2.49	2.46	2.68	2.62	2.52	2.53	2.64
Specific Strength (MY)	1.58	1.14	1.36	1.37	1.99	1.38	1.18	1.75	1.46	1.57
Specific Modulus (MY)	32.1	24.76	28.2	27.7	35.33	28.73	30.53	34.1	30.04	32.12
Component (wt %)	-	-	-	-	-	-	-	-	-	-
SiO_2_ Silica	100	75.5	71.8	65	65	61	60	60	55.2	51
Al_2_O_3_ Alumina	-	0.5	1	4	25	1	13.2	25	14.8	18
B_2_O_3_ Boron Trioxide	-	20	-	5	-	-	-	-	6.9	-
ZrO_2_ Zirconia	-	-	-	-	-	17	-	-	-	-
MgO Magnesia	-	0.5	3.8	3	10	-	3.1	6	3.3	7
CaO Calcia	-	0.5	8.8	14	-	2	22.1	9	18.7	5
Na_2_O Sodium Oxide	-	-	-	-	-	-	0.5	-	-	1
K_2_O Potash	-	3	13.6	8.7	-	16	0.6	-	0.3	6.4
LiO_2_ Lithium Oxide	-	-	0.6	-	-	3	0.2	-	0.2	4.5
F_2_ Fluorine	-	-	-	-	-	-	0.1	-	0.3	-
Fe_2_O_3_ Iron Oxide	-	-	0.4	0.3	-	-	0.2	-	0.3	6.1
MnO Manganese Oxide	-	-	-	-	-	-	-	-	-	0.3
H2O Water	-	-	-	-	-	-	-	-	-	0.4
P_2_O_5_ Phosphorous Oxide	-	-	-	-	-	-	-	-	-	0.3

**Table A6:** Composition of oxide ceramic fibers.

Oxide Ceramic Fibers	Type	Density (g/cm^3^)	Tensile Strength (GPa)	Specific Strength (MY)	E (GPa)	Es (MY)	Composition		
							Al_2_O_3_	SiO_2_	B_2_O_3_
3M Nextel 610 alumina silica	Crystalline	3.90	2.800	0.718	370.00	94.87	100%	-	-
3M Nextel 720	Crystalline	3.40	1.940	0.571	250.00	73.53	85%	15%	-
Nitivy ALF	Alumina-Silica	2.90	2.000	0.690	170.00	58.62	72%	28%	-
3M Nextel 550	Alumina-Silica	3.03	2.000	0.660	193.00	63.70	73%	27%	-
3M Nextel 440	Alumina-Silica	3.00	1.840	0.613	190.00	63.33	70%	28%	2%
3M Nextel 312	Alumina-Silica	2.80	1.630	0.582	150.00	53.57	63%	25%	13%
Sumitomo Altex	Alumina-Silica	3.30	1.800	0.545	210.00	63.64	85%	15%	-
Unifrax Saffil	Alumina-Silica	3.28	1.950	0.595	297.00	90.55	-	-	-

**Table A7:** Composition of nonoxide ceramic fibers.

Nonoxide Ceramic Fibers	Production Technique	Density (g/cm^3^)	Tensile Strength (Gpa)	Specific Strength (MY)	E (GPa)	Es (MY)	Composition					
							Si	C	O	Al	Zr	Ti
Nippon Hi-Nicalon S	Polycarbosilane	3.10	2.600	0.839	420.00	135.48	68.9%	30.9%	0.2%	-	-	-
Nippon Hi-Nicalon	Polycarbosilane	2.74	2.800	1.022	270.00	98.54	63.7%	35.8%	0.5%	-	-	-
Nippon Nicalon NL-200/201	Polycarbosilane	2.55	3.000	1.176	220.00	86.27	56.5%	31.2%	12.3%	-	-	-
UBE Industries Tyranno SA3	Polycarbosilane	3.10	2.800	0.903	380.00	122.58	67.8%	31.3%	0.3%	0.6%	-	-
UBE Industries Tyranno ZMI	Polycarbosilane	2.48	3.400	1.371	200.00	80.65	56.1%	34.2%	8.7%	-	1.0%	-
UBE Industries Tyranno LoxM	Polycarbosilane	2.48	3.300	1.331	187.00	75.40	55.4%	32.4%	10.2%	-	-	2.0%
UBE Industries Tyranno S	Polycarbosilane	2.35	3.300	1.404	170.00	72.34	50.4%	29.7%	17.9%	-	-	2.0%
Specialty Materials SCS Ultra SiC	CVD	3.08	5.900	1.916	415.00	134.74	SiC on carbon					
Specialty Materials SCS6 SiC	CVD	3.08	3.900	1.266	380.00	123.38	SiC on carbon					
Specialty Materials SCS-9A	CVD	2.80	3.450	1.232	307.00	109.64	SiC on carbon					
Tisics/Sigma	CVD	3.40	4.000	1.176	400.00	117.65	SiC on tungsten					
COI Ceramics/Sylramic-iBN	Precursor	3.00	3.000	1.000	400.00	133.33						
COI Ceramics/Sylramic	Precursor	2.95	2.700	0.915	310.00	105.08						
Avco 5.6mil SiC		3.07	3.792	1.235	413.69	134.75						
Avco 4.0mil SiC		3.30	3.792	1.149	413.69	125.36						
Nippon DC HVR Nicolon SiC		2.36	2.962	1.255	186.00	78.81						
Nicalon SiC		2.55	2.760	1.082	193.00	75.69						
Boron Carbide		2.00	2.758	1.379	379.21	189.61						
Boron Nitride		1.80	2.068	1.149	344.74	191.52						

**Table A8:** Composition of select metal fibers.

Metal Fibers	Density (g/cm^3^)	Tensile Strength (GPa)	Specific Strength (MY)	E (GPa)	Es (MY)	Composition				
						Fe	Ni	Cr	Mo	Co
AISI 316 Stainless	8.00	0.580	0.073	193	24.13	66	12	17	2	-
Inconel 601GC Nickel Superalloy	8.11	0.790	0.097	206.50	25.46	15	57	25	-	-
Haynes HR160 Nickel alloy	8.08	0.767	0.095	211	26.11	2	35	27	1	30
Hastelloy X alloy	8.22	0.755	0.092	205.00	24.94	18	47	22	9	1.5

## Appendix E. Production of Carbon Fibers from PAN

PAN fibers are converted into carbon fibers through a multiphase process. The first phase is known as oxidative stabilization. This phase takes place in air at 200 to 300 °C, typically 270 °C. At over 180 °C PAN molecular chains begin to unfold and move around. Five chemical reactions during this phase include: oxidation, dehydrogenation, cyclization, aromatization, and crosslinking.

1. Oxidation. In the oxidation reaction, oxygen atoms attach to the carbon chain, ejecting two hydrogen atoms in the form of N2 gas. The fibers incorporate approximately 8% oxygen during this exothermic process. Oxidation is the gain of oxygen atoms and loss of hydrogen atoms.
2. Hydrogenation. In the dehydrogenation reaction, double bonds are formed between carbon atoms to stabilize the carbon chain and eject oxygen and hydrogen atoms in the form of water vapor and N2 gas.
3. Cyclization. In the cyano group cyclization reaction, the C≡N triple bonds are broken, and formation of single and double bonds in a continuous ladder structure between alternating carbon and nitrogen atoms.
4. Aromatization. The aromatization reaction is the formation of a heterocyclic system created by cyclization of the nitrogen atoms.
5. Crosslinking. The crosslinking reaction is the bonding of one polymer chain to another. The structure results in aromatic pyridine groups as the carbon atoms lose hydrogen atoms and give off hydrogen gas. The crosslinking reaction sets the carbon structure.

The next phase after stabilization of PAN is the carbonization of PAN into carbon fiber. Carbonization is a pyrolytic reaction where organic compounds are converted into pure carbon in an exothermic process. This process takes place at 1000 to 1500 °C in an inert atmosphere, typically nitrogen. The optimal temperature for highest strength is 1400 to 1600 °C [60]. This process yields a turbostratic carbon fiber structure.

Polymer chains of stabilized PAN are joined together by the reduction of nitrogen.

Carbonization pushes the nitrogen molecules to the edge of the carbon structure. This results in carbon fiber.

If the carbon fiber is heated past 1500 °C, it will turn into graphite as the carbon sheets layer and join together. Graphitization is the heating of the fiber from 1500 to 3000 °C until the polymer contains 92% to 100% carbon. Pyrolysis is the thermal decomposition of materials in high temperature in an inert atmosphere. This process expels the impurities in the chain and removes the initial nitrogen molecules.

## Appendix F. Comparison of Specific Strength of Fiber Families

**Table A9:** Density, Yield Strength, and Specific Strength of Fiber Families.

Fiber Family	Density (g/cm^3^)	Yield Strength (GPa)	Specific Strength (MY)
Carbon Fibers	1.74 to 2.20	2.2 to 7.0	1.2 to 4.0
Intermediate Modulus Carbon Fiber (from PAN)	1.80 to 1.95	6.2 to 7.0	3.5 to 4.0
High Strength Carbon Fiber	1.74 to 1.80	3.5 to 4.9	2.0 to 2.7
High Modulus Carbon Fiber (from Pitch)	1.83 to 2.20	2.2 to 3.0	1.2 to 1.5
Polymer Fibers	-	-	-
Synthetic Polymer UHMW Polyethylene (Dyneema, Spectra, Tsunooga)	0.97 to 0.98	3.3 to 3.6	2.6 to 3.7
Synthetic Polymer PBO Polybenzoxazole (Zylon)	1.54	5.8	3.8
Synthetic Polymer, Polyester (Vectran)	1.4	2.9	2.21
Synthetic Polymer (Polyester, Polyimide, PVA)	1.39 to 1.43	0.52 to 1.85	0.37 to 1.32
Synthetic Polymer (Polypropylene, Nylon, Acrylic)	0.90 to 1.18	0.33 to 1.20	0.27 to 0.88
Aramid Polymer Fibers	-	-	-
Synthetic Polymer, Para Aramid (Kevlar)	1.44 to 1.47	2.8 to 3.8	1.9 to 2.6
Synthetic Polymer, Meta Aramid (Nomex)	1.38 to 1.38	0.3 to 0.6	0.3 to 0.5
Glass Fibers	-	-	-
Glass (A, C, E, and S type)	2.45 to 2.60	2.00 to 4.83	0.78 to 1.94
Ceramic Fibers	-	-	-
Nonoxides (Silicon Carbide, Silicon Nitride)	1.8 to 3.4	2.1 to 5.9	0.8 to 1.9
Oxides (Alumina)	2.8 to 3.9	1.4 to 2.8	0.4 to 0.7
Mineral Fibers	-	-	-
Basalt Fiber	2.64 to 2.70	2.5 to 4.8	1.0 to 1.8
Keratin Fibers	-	-	-
Spider Silk	1.31	1.40	1.07
Natural Wool	1.31	0.19	0.14
Regenerated Cellulose Fibers	-	-	-
Fortisan, Rayon, Tenasco, Acetate	1.25 to 1.52	0.17 to 0.89	0.13 to 0.59
Cellulose Fibers	-	-	-
Natural (Cotton, Silk, Flax, Jute, Hemp, Ramie)	1.27 to 1.54	0.29 to 0.89	0.19 to 0.59
Natural Wood fibers	0.14 to 0.74	0.07 to 0.9	0.12 to 0.52
Metallic Fibers	-	-	-
Steel, Aluminum	1.74 to 8.92	0.22 to 2.21	0.03 to 0.28
